# Bergerac strains of *Caenorhabditis elegans* revisited: expansion of Tc*1* elements imposes a significant genomic and fitness cost

**DOI:** 10.1093/g3journal/jkac214

**Published:** 2022-08-17

**Authors:** Austin T Daigle, Thaddeus C Deiss, Robert H Melde, Ulfar Bergthorsson, Vaishali Katju

**Affiliations:** Department of Veterinary Integrative Biosciences, College of Veterinary Medicine and Biomedical Sciences, Texas A&M University, College Station TX 77845, USA; Department of Veterinary Integrative Biosciences, College of Veterinary Medicine and Biomedical Sciences, Texas A&M University, College Station TX 77845, USA; Department of Veterinary Integrative Biosciences, College of Veterinary Medicine and Biomedical Sciences, Texas A&M University, College Station TX 77845, USA; Department of Integrative Biology, University of Wisconsin-Madison, Madison, WI 53706, USA; Department of Veterinary Integrative Biosciences, College of Veterinary Medicine and Biomedical Sciences, Texas A&M University, College Station TX 77845, USA; Department of Veterinary Integrative Biosciences, College of Veterinary Medicine and Biomedical Sciences, Texas A&M University, College Station TX 77845, USA; Program in Evolutionary Biology, Department of Ecology and Genetics (IEG), Uppsala University, 752 36 Uppsala, Sweden

**Keywords:** *Caenorhabditis elegans*, Bergerac, transposable element, transposon, copy-number variation, fitness, whole-genome sequencing, chromatin, recombination

## Abstract

The DNA transposon Tc*1* was the first transposable element to be characterized in *Caenorhabditis elegans* and to date, remains the best-studied transposable element in *Caenorhabditis* worms. While Tc*1* copy-number is regulated at approximately 30 copies in the laboratory Bristol N2 and the vast majority of *C. elegans* strains, the Bergerac strain and its derivatives have experienced a marked Tc*1* proliferation. Given the historical importance of the Bergerac strain in the development of the *C. elegans* model, we implemented a modern genomic analysis of three Bergerac strains (CB4851, RW6999, and RW7000) in conjunction with multiple phenotypic assays to better elucidate the (1) genomic distribution of Tc*1* and (2) phenotypic consequences of transposable element deregulation for the host organism. The median estimates of Tc*1* copy-number in the Bergerac strains ranged from 451 to 748, which is both (1) greater than previously estimated and (2) likely to be an underestimate of the actual copy-numbers since coverage-based estimates and digital droplet polymerase chain reaction results both suggest higher Tc*1* numbers. All three Bergerac strains had significantly reduced trait means compared with the N2 control for each of four fitness-related traits, with specific traits displaying significant differences between Bergerac strains. T*c1* proliferation was genome-wide, specific to Tc*1*, and particularly high on chromosomes V and X. There were fewer Tc*1* insertions in highly expressed chromatin environments than expected by chance. Furthermore, Tc*1* integration motifs were also less frequent in exon than noncoding sequences. The source of the proliferation of Tc*1* in the Bergerac strains is specific to Tc*1* and independent of other transposable elements. The Bergerac strains contain none of the alleles that have previously been found to derepress transposable element activity in *C. elegans*. However, the Bergerac strains had several Tc*1* insertions near or within highly germline-transcribed genes which could account for the recent germline proliferation.

## Introduction

Transposable elements (TEs henceforth) are small DNA fragments particularly ubiquitous in eukaryotic genomes that occasionally relocate to new genomic positions, hence the moniker of “jumping genes” (McClintock 1950; Chuong *et al.* 2017). TEs are viewed as parasitic and selfish because they rely on the host genome’s cellular machinery to mobilize, and their ability to spread despite deleterious fitness costs associated with disruption of host gene function due to insertions (Orgel and Crick 1980; Muñoz-López and García-Pérez 2010). As such, both prokaryotic and eukaryotic host genomes have evolved complex surveillance and regulatory systems for TE detection and silencing (Sijen and Plasterk 2003; Blumenstiel 2011; Klein and O'Neill 2018; Payer and Burns 2019). Despite their overwhelmingly deleterious effects for host genomes, these mobile genetic elements can also inadvertently contribute to novel advantageous phenotypes as documented in the rapid phenotypic evolution observed in the British peppered moth (van’t Hof *et al.* 2011), the role of TEs in gene transcription as *cis*-regulatory elements in many organisms (Rebollo *et al.* 2012) and their domestication as a host recombinase mechanism required for genomic rearrangements integral to the adaptive immune systems of higher vertebrates (Zhang *et al.* 2019). Recent research has utilized increasingly sophisticated computational tools and a comparative genomics framework to understand the broad genomic effects and functions of TEs (Bourque *et al.* 2018) while emphasizing the need for studies focusing on the initial proliferation of diverse TE families in diverse taxa (Wells and Feschotte 2020). To fully understand the coevolution between TE regulation and expansion in evolutionary diversification, the mechanisms by which TEs evade regulation at the expense of the host organism requires further elucidation.

The nematode *Caenorhabditis elegans* has been a useful model for TE research since the discovery of the first *C. elegans* TE, a DNA transposon named Tc*1* (Emmons *et al.* 1983). Tc*1* and its homologs are among the most abundant TEs occurring in nature (Plasterk *et al.* 1999). Overall, TEs comprise approximately 12% of the genome of the canonical wild-type Bristol N2 strain (*C. elegans* Sequencing Consortium 1998). A recent study of TEs in the genomes of 208 *C. elegans* wild isolates revealed significant variation in the copy-number of diverse TE classes (Laricchia *et al.* 2017), highlighting the contribution of TEs to intraspecific genome variation. Transcription of TEs is under strong purifying selection in *C. elegans* (Bergthorsson *et al.* 2020). The primary mechanisms responsible for the regulation of transposons and other foreign DNA in the *C. elegans* genome are composed of small RNAs (sRNAs), including 21U-RNAs, 26G-RNAs, and 22G-RNAs (Ambros *et al.* 2003), that couple with and guide Argonaute (AGO) proteins (Yigit *et al.* 2006). Nematode sRNAs have distinct structures and biogenesis pathways, but often share common regulatory functions (Almeida *et al.* 2019). 21U-RNAs, a special class of sRNA known as PIWI-interacting or piRNAs, were determined to interact directly with the AGO protein *prg-1* to prevent TE spread in germ line cells (Batista *et al.* 2008; Bagijn *et al.* 2012). Despite evidence that 21U-RNAs play a role in controlling TE transcription, only the mariner transposon *Tc3* has been demonstrated to become mobilized upon disruption of piRNA machinery (Das *et al.* 2008; Reed *et al.* 2020). High-throughput sequencing of piRNAs and siRNAs in various mutants seemed to suggest that WAGO-class 22G-RNAs played a larger role in transposon silencing, and it had been suggested that the mutants studied could have inherited long-term effects of piRNA silencing due to multigenerational epigenetic memory (Ashe *et al.* 2012; Reed *et al.* 2020). However, a recent experimental study in *C. elegans* demonstrated that 22G-RNA epimutations are typically unstable with an average persistence time of 2–3 generations (Beltran *et al.* 2020). Many details and overlapping features of the intricate regulatory pathways involved in silencing TEs and other germline transcripts in *C. elegans* await further elucidation.

The Bergerac strain of *C. elegans* is of historical importance in the development of the species as a model organism (Riddle *et al.* 1997). Bergerac was one of the earliest laboratory strains of *C. elegans* to be cultured and was isolated from garden soil in 1944 in Bergerac, France by Victor Nigon of the Université de Lyon (Nigon 1949; Nigon and Félix 2017). Some of the earliest genetic work in *C. elegans* occurred with the use of the Bergerac strain, including the discovery of a Mendelian temperature-sensitive allele (Fatt and Dougherty 1963). Owing to difficulties with propagation later determined to be due to temperature-sensitivity of hermaphrodites (Fatt and Dougherty 1963), infertile males (Fatt and Dougherty 1963), and high mutation rates (Moerman and Waterston 1984), the use of the Bergerac strain for subsequent genetic work was abandoned and replaced by the Bristol N2 strain isolated by L. N. Staniland from a mushroom compost heap in Bristol, England (Nicholas *et al.* 1959; Brenner 1974; Riddle *et al.* 1997; Nigon and Félix 2017). Several decades later and with a more developed arsenal of molecular and genetic tools available, several *C. elegans* researchers took to investigating the mechanistic bases of some of these aberrant phenotypes associated with the Bergerac strain. Bristol N2 and Bergerac were observed to exhibit differences in restriction endonuclease cleavage patterns on Southern hybridizations when probed with randomly selected cloned fragments (Emmons *et al.* 1979, 1983; Files *et al.* 1983). Southern hybridization analyses further demonstrated that while Bristol N2 possessed 20 ±5 dispersed copies of Tc*1* in the genome, the Bergerac strain appeared to have an estimated 200 ± 50 copies (Emmons *et al.* 1983). Further investigation of Tc*1* copy-number in 10 newly available *C. elegans* isolates led Emmons *et al.* (1983) to the parsimonious conclusion that these intraspecific differences in transposon copy-number were owing to the uniquely massive proliferation of Tc*1* elements in the Bergerac strain that likely resulted in gene disruption leading to phenotypic defects. A later study using quantitative dot blot hybridization presented estimates of Tc*1* copy-number in the Bristol N2 and Bergerac strain as being ~ 30 Tc*1* and 300–550 copies, respectively (Egilmez *et al.* 1995). Rosenzweig *et al.* (1983a) were the first to generate the complete 1,610-bp nucleotide sequence of the Tc*1* TE in *C. elegans* with its characteristic short inverted terminal repeats of <100 bp. Liao *et al.* (1983) provided a substantially detailed characterization of this *C. elegans* Tc*1*, namely that (1) while it shared many structural features with other eukaryotic DNA TEs, it belonged to a unique class, and (2) all *C. elegans* Tc*1* copies appeared to display full-length conservation unlike *Drosophila P*-elements, suggesting that it encodes products mediating its own transcription. Tc*1* elements in the Bergerac strain were found to be significantly more active relative to their counterparts in the Bristol N2 genome, displaying site-specific insertion and excision from the muscle gene *unc-54* (Eide and Anderson 1985, 1988). In addition to increased transposition, it was noted that Bergerac strains were less fit, produced fewer progeny, moved with less coordination, and had a higher incidence of males relative to other *C. elegans* strains, despite the males being sterile (Hodgkin and Doniach 1997). It has been hypothesized that the increase in Tc*1* copy-number occurred in the laboratory after Nigon isolated Bergerac from the wild (Moerman and Waterston 1984; Egilmez *et al.* 1995; Nigon and Félix 2017). However, the cause of the massive proliferation of Tc*1* elements in the Bergerac strains remains to be identified.

While some traits in Bergerac have been studied, mainly in the strain RW7000 (Fatt and Dougherty 1963; Shook and Johnson 1999; Vertino *et al.* 2011; Lee *et al.* 2016), a comparative study of multiple fitness-related traits and precise quantification of composite fitness has yet to be conducted on multiple distinct Bergerac strains simultaneously. This project is the first to employ high-throughput Illumina whole-genome sequencing technology to sequence and analyze the entire genomes of three distinct Bergerac strains. While the genome of one Bergerac strain, CB4851, has previously been sequenced, it has not yet been analyzed to study Tc*1* proliferation (Cook *et al.* 2017; Laricchia *et al.* 2017). Herein, we quantified and compared four fitness-related traits (developmental rate, productivity, longevity, and survivorship) in order to discern extant phenotypic variation among the Bergerac strains RW6999, RW7000, and CB4851. Next, using whole-genome sequencing and digital droplet polymerase chain reaction (ddPCR) data for each strain, we generate more accurate TE copy-number estimates, quantify the decreased and variable fitness across Bergerac strains relative to Bristol N2, analyze the distribution and sequence context of Tc*1* landing sites, and conduct an initial search for the cause of increased Tc*1* proliferation.

## Materials and methods

### Bergerac strains of *C. elegans* used in this study

This study focused on three subclones of the original Bergerac strain isolated by Nigon in Bergerac, France in 1944 (Nigon and Félix 2017). This strain was shared with several other laboratories before the implementation of cryopreservation techniques for *C. elegans*, and hence the names of these subclones of the original Bergerac strain either represent their initial culture location or standardized strain nomenclature that came to be adapted later. We focused on three Bergerac strains to quantify their fitness declines and genomic divergence that may have occurred during laboratory evolution and divergence following the proliferation of Tc*1* elements. The first, RW7000 (also known as Bergerac BO for its location in Boulder, CO, USA), belongs to the Boulder BO sublineage and was given to David Hirsh by Nigon’s student Jean-Louis Brun in 1983, and used in many of the original studies of TEs in *C. elegans* (Liao *et al.* 1983; Rosenzweig *et al.* 1983a; Moerman and Waterston 1984; Mori, Moerman *et al.* 1988; Nigon and Félix 2017). A second Bergerac strain, RW6999, is relatively understudied and is a subclone of RW7000 as per the Caenorhabditis Genetics Center (CGC) (https://cgc.umn.edu/strain/RW6999). A third strain, CB4851, belonging to the Cambridge sublineage (Nigon and Félix 2017), was shared with Sydney Brenner in 1969 (Hodgkin and Doniach 1997; Nigon and Félix 2017). Our rationale for selecting these three strains was two-fold, in aiming to investigate how TE distribution varies (1) between laboratory isolates of both recent and more distant ancestry and (2) between active mutator strains (RW6999 and RW7000; Moerman and Waterston 1984; Eide and Anderson 1985) and one lacking active germline transposition (CB4851; Nigon and Félix 2017).

### Fitness assays for four life-history traits and statistical analyses of fitness data

To quantify the fitness of Bergerac strains RW7000, RW6999, and CB4851 relative to the laboratory strain Bristol N2 (also referred to as N2), we assayed four fitness-related, life-history traits, namely (1) productivity, (2) survivorship to adulthood (also referred to as survivorship), (3) longevity, and (4) developmental rate as previously described (Katju *et al.* 2015, 2018; Dubie *et al.* 2020). All assays were conducted on Nematode Growth Medium (NGM) agar plates seeded with the *E. coli* strain OP50 at 20°C, the standard temperature for *C. elegans* culturing. Cryopreserved stocks of RW7000, RW6999, and CB4851 and N2 were thawed and individual worms isolated onto NGM plates. In order to establish independent lines, 15 and 20 worms were isolated for N2 and each Bergerac strain, respectively. After the establishment of multiple lines per strain, the worms were allowed to self and five of their L4 larval stage progeny were isolated singly onto a new plate, thereby establishing five sublines per line (total 15 × 5 = 75 for N2; 20 × 5 = 100 for each Bergerac strain). To negate the possibility of maternal or grandmaternal effects (Lynch 1985), each replicate was propagated by single-worm transfer for one more generation. This hierarchical structure (strains, lines, and sublines) combined with the fact that hermaphrodites self-fertilize to produce the next generation, minimizes genetic divergence among replicates, and allows for a clean estimate of environmental variance by comparison of sublines. Three fitness assays (development, productivity, and longevity) were conducted on a single, third-generation worm isolated from each subline as an L1 larva. To measure the developmental rate, commencing 36 h after the initial isolation of the L1 larva, each worm was checked every 2 h to identify the time (in hours) until the first egg was visible in the worm’s uterus. Upon noting the presence of the first egg in the uterus, the worm was scored as having developed to adulthood. This initial measurement of the time (hours) from L1 larva to an egg-bearing adult yielded the developmental time. The inverse of the developmental time yielded the developmental rate. Worms that died before reaching adulthood were not scored.

To assay productivity, each worm that developed to adulthood was transferred to a new plate every 24 h for eight consecutive days. After transferring the worm to a new plate, the eggs from the previous plate were allowed to hatch for an additional 24 h, then stored at 4°C for a minimum of one month to allow the progeny to die without producing offspring. Counts were conducted by staining each plate with a 0.075% water dilution of toluidine blue, which temporarily makes the progeny stand out white against a purple background to facilitate visualization for worm counts.

Following eight days of daily transfers to a fresh plate as part of the productivity assay, each worm was transferred to a fresh NGM agar plate seeded with the *E. coli* OP50 until death in order to score longevity in days. In order to score longevity, the worms were monitored each day for movement and pharyngeal pumping. When no movement was detected, the agar pad near the worm was gently tapped. If no response was detected, the tail of the worm was tapped. If the worm failed to respond, it was recorded as dead and the days to mortality were noted.

To assay survivorship to adulthood, we used 10 L1 siblings of each third-generation worm assayed for the preceding three life-history traits. The 10 L1 larvae were isolated onto a seeded 60 mm NGM agar plate. For some sublines, less than 10 L1 individuals were isolated due to the low and delayed productivity of Bergerac worms. Thirty-six hours after isolation, the plates were checked for worms that survived to adulthood, and each plate was scored using the fraction of worms that survived to adulthood (values ranged from 0 to 1). For plates with desiccated worms on the edge of the plate, worms were still scored as surviving if eggs were observed in the uterus, to provide a more conservative estimate of survivorship.

For each of the four fitness traits, a two-level nested Model I ANOVA with unequal sample sizes (Sokal and Rohlf 1995) was employed to partition the total phenotypic variance into among- and within-line components. The highest level of classification tested for a treatment effect (four strains as treatments): (1) the laboratory wild-type strain N2, (2) strain RW7000, (3) strain RW6999, and (4) strain CB4851. The next level of hierarchy tested for a subgroup variance component (difference among independent lines within a treatment). The last hierarchical level estimated the subline variance. To conduct pairwise comparisons for all strain pairs per fitness trait, a Tukey–Kramer HSD test for unequal sample sizes was used at a 5% experiment-wise error rate.

### Motility- and size-associated assays

In addition to the fitness assays, we further quantified trait means for (1) speed, (2) body length, (3) body area, and (4) direction change in the Bergerac strains relative to the laboratory strain N2. Cryopreserved stocks of each strain were thawed and bottlenecked for three generations to establish five replicates for each strain, using procedures similar to the previously described fitness assays. Two identical assays were conducted on separate days, resulting in recordings of 10 plates for each strain in total. When the progeny of each third-generation replicate reached the L4 life stage, 10 hermaphrodite siblings were isolated onto a single 35 mm × 100 mm plate with 3.25 ml NGM; 21–22 h after isolating L4s for each strain, the young adult worms were removed from a 20°C incubator and transferred to an unseeded NGM plate. Within 3 min of the transfer, a 60-s video was recorded using Zen on an Axio Zoom.V16 Zoom microscope equipped with an AxioCam 503 Color camera at 5 fps. The objective lens used for this analysis was the Zeiss PlanNeoFluar Z 1×/0.25 FWD 56 mm. The conversion factor for videos provided by the Zen program was 25.94 µM/pixel. The resolution of the final videos was 968 × 730. The order we recorded strains was randomized, and worms were kept in the incubator as long as possible to limit environmental variance. The temperature in the recording room was recorded at least three times for each assay and remained at approximately 21 ± 1°C. These details are provided to increase the reproducibility of our motility assay in a manner consistent with the suggestions of Angstman *et al.* (2016).

A custom Matlab program called Zentracker was used to analyze assay videos (https://github.com/wormtracker/zentracker). After inputting the scaling factor provided by the scope, all videos were tracked at intensities ranging from 130 to 180, then manually validated to ensure all worms were tracked and nonworm objects were removed. After checking the validity, the values for average speed, length, area, and direction change were extracted for each video. In this analysis, the trait mean represents the average of 10 worms on a plate for 1 min. A one-level ANOVA test was used to reveal significant differences for each trait measured (speed, direction, body length, and body area). We additionally conducted comparisons among pairs of means using the Tukey–Kramer HSD method to determine which strain pairs differed significantly from each other for the four motility- and size-associated traits.

### DNA extraction and Illumina sequencing

Genomic DNA was extracted from Bergerac and N2 control lines as previously described (Konrad *et al.* 2018) with libraries prepared using the Nextera DNAflex library kit (Illumina, San Diego, CA, USA). Libraries were sequenced on the Illumina Novaseq6000 platform (2 × 150 bp) at the North Texas Genome Center at the University of Texas at Arlington.

### Tc*1* copy-number estimation with ddPCR

A ddPCR copy-number variation (CNV) assay was performed following the Bio-Rad ddPCR Copy-Number Variation Assay protocol (https://www.bio-rad.com/webroot/web/pdf/lsr/literature/10033173.pdf) for lines N2, CB4851, RW6999, and RW7000. The ddPCR utilizing a Tc*1* targeting probe and a *daf-3* (a single-copy reference) targeting probe with Fluorescein (FAM) were analyzed on the Bio-Rad QX ONE ddPCR system. The haploid genome-wide copy-number of Tc*1* was determined based on the estimated copy-number of Tc*1*-positive relative to the *daf-3* single-copy control. The reactions were run using two concentrations of template genomic DNA: (1) the first, at 0.1 ng/µl, to allow a sufficient number of *daf-3* positive droplets, and (2) another, a 100-fold dilution of 0.001 ng/µl, to avoid saturation of positive Tc*1* droplets. The number of *daf-3* positives was then divided by the dilution factor to identify the proportion of negative droplets for Poisson calculations shown below. The cutoffs for positive and negative droplets were assigned manually on the QX Manager Software (1.2 Standard Edition) provided by Bio-Rad based on the separation of the droplets.

### Tc*1* copy-number estimation from whole-genome sequence data

Tc*1* copy-number in each genome was also estimated using the McClintock meta-pipeline, v2.0.0 (Nelson *et al.* 2017) which combines many TE-detection algorithms to identify reference and nonreference TE insertions in each genome. A standard consensus sequence of a 1,610-bp Tc*1* element was used as an input. Each TE-detection algorithm produces unique results containing both correct calls and false positives, with varying degrees of sensitivity and precision in simulated and real datasets (Nelson *et al.* 2017; Vendrell-Mir *et al.* 2019). McClintock v2.0.0 provides an estimate of normalized mean coverage. Five callers were chosen from the McClintock pipeline to estimate Tc*1* copy-number based on the absolute difference from the normalized mean coverage and ddPCR copy-number estimate: ngs_te_mapper2 (Han *et al.* 2021), RelocaTE (Robb *et al.* 2013), TEMP2 (Yu *et al.* 2021), RetroSeq (Keane *et al.* 2013), and TEFLoN (Adrion *et al.* 2017).

To visualize the overlap between the results of the various McClintock callers, the reference genome (PRJNA13758.WS279) was split into 1,000-bp windows using BEDTools (Quinlan and Hall 2010). The BEDTools “intersect” command was used to create a file with a window for each nonreference Tc*1* call from each McClintock caller. Given that the reference Tc*1* elements tend to span several 1,000-bp windows, a similar procedure was used to assign reference calls to 100,000-bp windows, and subsequently these files were combined with the nonreference windows. Using a web Venn diagram tool (http://bioinformatics.psb.ugent.be/webtools/Venn/), Venn diagrams describing the overlap of Tc*1* calls for each method in each strain were produced, along with a Venn diagram describing the overlap of TEMP2 Tc*1* calls between the Bergerac strains.

In addition to Tc*1*, we employed the TEMP2 caller from McClintock to estimate copy-number for other TEs, namely Tc*2*, Tc*3*, *Tc4v*, *Tc5*, *Tc6*, *Tc9*, *TURMOIL1*, *TURMOIL2*, *MARINER2*, *MARINER3*, *MARINER4*, and *MARINER5*.

### Tc1 landing site sequence analysis

Due to the high positional accuracy of the TEMP2 caller (_**∼**_0.90) in the prediction of synthetic TE insertions used as benchmarks in the original McClintock publication, it was chosen for analyses of TE landing sites (Nelson *et al.* 2017). The sequence context of each Tc*1* insertion predicted by TEMP2 was analyzed in each Bergerac strain. The FASTQ reads generated by Illumina whole-genome sequencing were aligned to the *C. elegans* reference genome (PRJNA13758.WS279) with the Burrows–Wheeler Aligner (BWA), version 0.7.12-r1039. Using the BED file generated by the McClintock pipeline, sequences _**±**_25 bp from each insertion site were extracted from the BWA alignments. These sequences were aligned using ClustalW and a consensus sequence was generated for each strain with IUPAC nucleotide code by analyzing the distribution of nucleotides _**±**_6 bp from the center of the insertion.

### Statistical analyses of TE distribution in the Bergerac genomes

To assess the environment of Tc*1* landing sites within the Bergerac strains’ genomes, genomic coordinates specific to various genomic features were used to define the genomic context of Tc*1* insertions. Annotation files were created for chromosomal arms, cores, and tips, exon, intron, or intergenic regions, as well as various histone modification environments. To preclude overlapping regions with the annotation files from yielding multiple counts, these annotation files were then merged to prevent overlapping regions within the annotation files from yielding multiple counts using the “merge” command of the BEDTools software package (Quinlan and Hall 2010). Finally, the merged BED files denoting the landing site environment and the resulting Tc*1* BED files for each line were analyzed for overlap using the bedtools “intersect” command. For exon and intron overlap with identified Tc*1* sites, the merged exon and intron annotations were analyzed for intersections with the “-v” flag ensuring only intron exclusive genomic sites were used to call intronic sites. The resulting counts were used as input for chi-squared tests, using the proportion of genomic coverage to calculate expected values within the R statistical analysis software (R Core Team 2014).

### Genes with exons disrupted by Tc1

Using the *C. elegans* N2 reference annotations file from WormBase (version WS279; www.wormbase.org), a table of genes with predicted exonic or intronic Tc*1* insertions according to TEMP2 was generated along with a list of intergenic insertions. Some insertions in this table are repeated, as the exons of genes with multiple transcripts do not always agree. Using the WormBase tool SimpleMine (https://wormbase.org/tools/mine/simplemine.cgi), a table containing a description of RNAi phenotypes, allele phenotypes, concise descriptions, and automated descriptions for genes disrupted by Tc*1* was created.

### Analysis of SNPs and CNV in the Bergerac strains

In order to identify candidate mutations responsible for Tc*1* proliferation in the Bergerac strains, the MiModd package (www.celegans.de/mimodd) was used to identify SNPs, indels, and deletions in the 3 Bergerac strains, the N2 strain housed in the laboratory, and 7 additional natural isolates: AB1, ECA243, JU1568, JU2565, JU394, NIC1049, and NIC2. One isolate, ECA243, is a renamed sample of the strain CB4851 that was previously sequenced. The BAM alignments for the 7 chosen natural isolates were obtained from the CeNDR database, release 20210121 (Cook *et al.* 2017). The reads for the Bergerac strains and our laboratory sample of N2 were cleaned with Fastp using default settings and aligned with BWA–MEM (BWA maximal exact match) to the *C. elegans* reference genome PRJNA13758.WS276 to replicate the CeNDR procedure. All 11 BAM files were then merged, and MiModd was used to extract SNPs and indels for all strains. These variants were filtered to keep homozygous variants with a minimum depth of 3. SNPeff, a tool to predict the effect of SNPs on protein-coding genes, was used to annotate these variants (Cingolani *et al.* 2012). In addition to SNPeff, SIFT 4G was used to predict which amino acid changes in the Bergerac strains are likely to be deleterious to protein function. Deletions were also called for all strains using MiModd, with a maximum coverage of 4 and a minimum size of 100 bp. After calling variants and deletions, the mutations were filtered to retain homozygous variants identified in all 3 Bergerac strains and absent in all non-Bergerac strains. Genes with deletions were determined using the *C. elegans* WS276 reference gff3 file. Summary tables for all mutated genes were created using SimpleMine. Finally, to place the Bergerac strains in a phylogenetic context, we ran a maximum likelihood analysis with PhyML (Guindon *et al.* 2010), using a GTR substitution model with a discrete gamma distribution and 4 rate categories on 11 *C. elegans* strains, which included the 3 Bergerac strains and N2.

## Results

### Significant variation in Tc1 copy number among the Bergerac strains

**Fig. 1.**
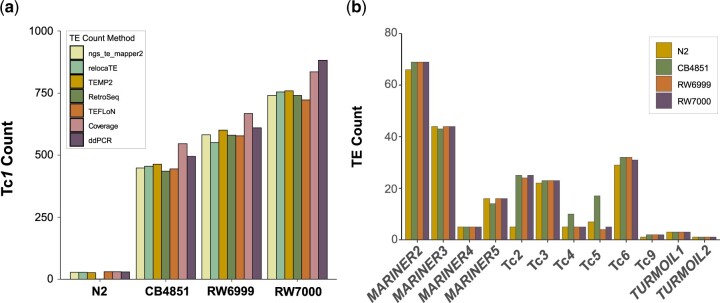
Proliferation of the Tc*1* transposon in the Bergerac strains of *C. elegans*. a) Counts of the Tc*1* transposon in our laboratory isolate of Bristol N2 and 3 Bergerac strains, CB4851, RW6999, and RW7000 using various computational and molecular methods. Computational methods included were ngs_te_mapper, RelocaTE, TEMP2, RetroSeq, TEFLoN, and coverage. Copy-number estimation via ddPCR was the sole molecular method. b) Counts of other/non-Tc*1* transposable elements are displayed for N2, CB4851, RW6999, and RW7000. Tc*2* was the only transposable element with a marked increase in the 3 Bergerac lines compared with N2, while Tc*4* and Tc*5* were found to be marginally higher in CB4851.

The median and average number of Tc*1* elements in our reference Bristol N2 was 28, with a range of 26–30 copies based on 5 methods (ngs_te_mapper 2, RelocaTE, TEMP2, Teflon, Normalized TE coverage) implemented in the McClintock pipeline (Fig. 1a and Table 1; Supplementary Table 1). The RetroSeq caller of the McClintock program does not call reference TEs; hence an estimate was omitted for the N2 strain. ddPCR results from our N2 control estimated the Tc*1* copy-number as 29.

**Table 1.** Estimated number of Tc*1* transposable elements in the reference N2 and 3 Bergerac strains of *C. elegans*.

*CAENORHABDITIS ELEGANS* STRAIN	ESTIMATES OF GENOMIC Tc*1* COPY-NUMBER BY MCcLINTOCK					NORMALIZED Tc*1* COVERAGE	RANGE	MEAN	MEDIAN	DDPCR
	ngs_te_mapper 2	RelocaTE	TEMP2	RetroSeq^*a*^	TEFLoN					
N2	28	28	26	–	30	30	26–30	28	28	29
CB4851	448	455	463	435	444	546	435–546	465	452	455
RW6999	582	551	601	580	578	668	551–668	593	581	610
RW7000	741	755	759	741	723	836	723–836	759	748	882

The haploid number of Tc*1* as determined by several TE element callers in the McClintock pipeline (Nelson *et al.* 2017). The range, means, and medians are calculated from estimates provided by 6 selected callers in the McClintock pipeline. Copy-number estimates of Tc*1* from ddPCR are included for comparison.

^a^ The RetroSeq caller of the McClintock program does not call reference TEs; hence, an estimate was omitted for the N2 strain.

The 3 Bergerac strains display substantial variation in Tc*1* copy-number that is consistent between different variant callers notwithstanding some variation in results between different methods (Fig. 1a and Table 1). The range in Tc*1* copy-number estimates among the Bergerac strains was nonoverlapping (Table 1). CB4851 harbored the lowest number of Tc*1* elements with a mean and median of 465 and 451, respectively. The mean and median of Tc*1* copy-number in RW6999 was 593 and 581, respectively. RW7000 has the highest abundance of Tc*1* elements with a mean and median estimate of 759 and 748, respectively (Fig. 1a and Table 1). The estimates based on normalized Tc*1* coverage were 12–21% greater than those based on the 5 methods that use paired-ends and split reads to estimate copy-number. BED files containing the locations of all calls produced by McClintock are provided in Supplementary Table 1. ddPCR estimates of Tc*1* copy-number in the 3 Bergerac strains were consistently higher than their corresponding median copy-number estimates from McClintock, ranging from 495, 610, and 882 copies for CB4851, RW6999, and RW7000, respectively. Both ddPCR and McClintock estimates support the general conclusion that the 3 Bergerac strains can vary considerably with respect to Tc*1* copy-number.

**Fig. 2.**
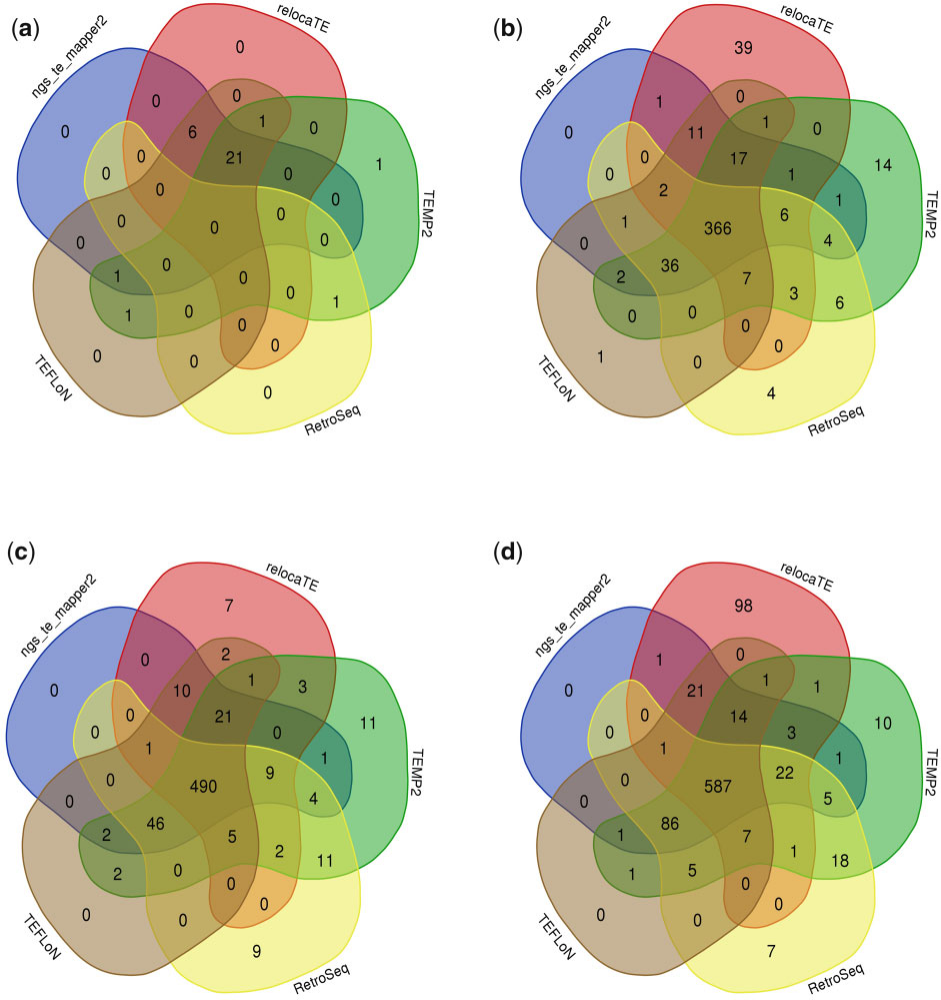
Comparison of Tc*1* insertions predicted by McClintock component methods. The positional agreement of the McClintock component methods was assessed by assigning Tc*1* calls to windows (1,000- and 100,000-bp windows for nonreference and reference calls, respectively) followed by a comparison of the calls made by each component method. The Venn diagrams compare the agreement of Tc*1* calls for a) N2, b) CB4851, c) RW6999, and d) RW7000. Computational methods included were ngs_te_mapper2 (blue), RelocaTE (red), TEMP2 (green), RetroSeq (yellow), and TEFLoN (brown). The vast majority of Tc*1* insertions were called by multiple methods.

To assess whether McClintock component methods were calling Tc*1* insertions in similar locations, Tc*1* calls produced by ngs_te_mapper2, RelocaTE, TEMP2, RetroSeq, and TEFLoN were assigned to 1,000_**-**_bp windows and compared (Fig. 2). The vast majority of Tc*1* calls are supported by multiple callers, with 70% of the 523 unique Tc*1* calls in CB4851 supported by all 5 callers, and 83% of calls supported by 4 or more callers. In addition to the comparison of Tc*1* insert locations between callers, the overlap of insert locations between the Bergerac strains was assessed by comparing the locations of calls produced by TEMP2 (Supplementary Fig. 1). Though a fraction of insert sites are shared between all 3 strains, the Bergerac strains display high variability in the location of insert sites, with large differences observed between the strain CB4851 and the 2 RW strains. This pattern reflects continued Tc*1* mobility and proliferation during the separate laboratory cultivations of these strains reported by Nigon and Félix (2017), with the RW strains sharing a more recent common ancestor.

### Variation in non-Tc1 elements in the Bergerac strains

The McClintock package was also used to estimate the copy-number of a wider variety of TEs in the 3 Bergerac strain relative to N2 (Supplementary Table 1). There were no differences in the estimated counts between N2 and the Bergerac strains for the majority of non-Tc*1* elements (*Mariner2-5*, Tc*3*, Tc*6*, Tc*9*, *Turmoil1*, and *2*) (Fig. 1b). However, the Bergerac strains appear to have more Tc*2* elements than N2. The average estimated count of Tc*2* was 3 and 20 in the N2 and the Bergerac strains, respectively. Coverage estimates using normalized read depth ranged between 25 and 30 in the Bergerac strains and 4 in N2. The average counts of Tc*4* and Tc*5* appeared to be higher in CB4851 than in the other 2 Bergerac strains (RW6999 and RW7000) and N2. However, the results were highly variable between different callers in McClintock, especially for Tc*5* where the number of counts ranged from 0 to 40, and the distribution of counts exhibited considerable overlap between the 4 strains. Furthermore, normalized read depth did not provide support for differences in Tc*5* counts between the strains, with estimates of 13–14 elements per strain.

### Bergerac strains have significantly lower fitness than the N2 strain

**Fig. 3.**
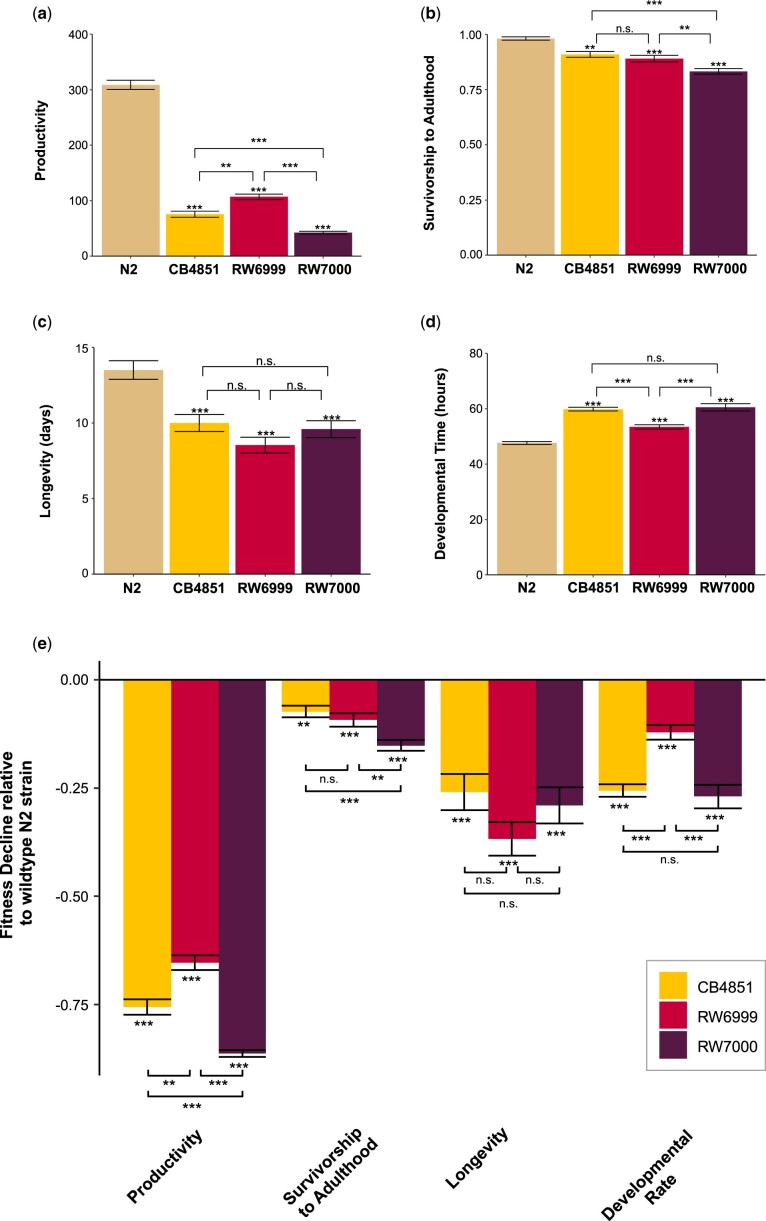
Significant fitness reduction in Bergerac strains relative to N2. Means for 4 fitness-related traits, namely a) productivity, b) survivorship to adulthood, c) longevity, and d) developmental time observed in the N2 control, and the 3 Bergerac strains CB4851, RW6999, and RW7000. e) Decline in mean relative fitness of the 3 Bergerac strains relative to the N2 strain. For simplicity, the mean relative fitness value for each of the 4 traits in the Bristol N2 control was scaled to a value of 1 (not displayed). For all panels, error bars represent 1 SE. Significance was determined via ANOVA and is displayed as asterisks where *P ≤ *0.05*/0.01**/0.001***. Exact *P*-values for the pairwise strain comparisons using Tukey–Kramer HSD can be found in Supplementary Tables 2–5.

We empirically quantified the fitness of the 3 Bergerac strains (CB4851, RW6999, RW7000) relative to the Bristol N2 strain by measuring 4 fitness traits, namely productivity, survivorship to adulthood, longevity, and developmental time. The mean fitness values for each measured trait are displayed in Fig. 3, a–d and Table 2.

**Table 2.** Fitness of 3 *C. elegans* Bergerac strain relative to worms of the laboratory strain, N2.

*CAENORHABDITIS ELEGANS* STRAIN	PRODUCTIVITY	SURVIVORSHIP TO ADULTHOOD (%)	LONGEVITY (DAYS)	DEVELOPMENTAL TIME (H)
N2	308.7 (8.4)	98.1 (0.8)	13.5 (0.6)	47.6 (0.6)
CB4851	75.5 (5.5)	90.9 (1.3)	10.0 (0.6)	59.8 (0.8)
RW6999	106.9 (5.1)	89.0 (1.5)	8.5 (0.6)	53.4 (0.9)
RW7000	42.3 (2.4)	83.2 (1.3)	9.6 (0.7)	60.5 (1.5)

For N2, 15 lines each with 5 replicates were established (maximum *n *=* *75). For each of the 3 Bergerac strains (CB4851, RW6999, and RW7000), we assayed 20 lines with 5 replicates each (maximum *n *=* *100). Estimates of the mean phenotype and 1 SE (in parentheses) for 4 fitness-related traits are provided for each of the 4 strains.

The Bergerac strains exhibited a 65–86% reduction in mean productivity relative to the N2 strain (Table 2 and Fig. 3, a and e). As reported in a previous study of Bergerac-derived recombinant inbred lines (Shook and Johnson 1999), many Bergerac worms were observed to die by matricidal hatching (“worm bagging” or “bagging”), a phenotype characterized by larvae hatching within the hermaphrodite before egg-laying occurs. However, these worms were observed to lay some eggs before bagging and were included in the analysis to provide a realistic estimate of mean productivity in laboratory conditions. ANOVA analyses found a significant variance component for productivity among the 4 strains (*F*_S_′ = 309.93; *P < *0.0001) whereas among-line divergence was nonsignificant (Table 3).

**Table 3.** Two-level nested ANOVA for productivity, survivorship to adulthood, longevity, and developmental time of N2 and Bergerac strains CB4851, RW6999, and RW7000.

Source of variation	*df*	SS	MS	*F*_s_	*F*_s_′
Productivity					
Among groups	3	3,069,207	1,023,069	403.83	309.93****
Among lines	69	217,546	3,153	1.25	
Within lines (error)	224	567,480	2,533		
Total	296				
Survivorship to adulthood					
Among groups	3	0.945	0.315	23.88	16.80****
Among lines	69	1.289	0.019	1.42*	
Within lines (error)	274	3.615	0.019		
Total	346				

Longevity					
Among groups	3	1,044	348.0	12.77	12.49****
Among lines	68	2,029	29.8	1.10	
Within lines (error)	220	5,995	27.3		
Total	291				

Developmental time					
Among groups	3	7,804	2,601.3	35.51	34.06****
Among lines	69	5,348	77.5	1.06	
Within lines (error)	223	16,337	73.3		
Total	295				

* Significance level of 0.05.

** Significance level of 0.01.

*** Significance level of 0.001.

**** Significance level of 0.0001.

The Bergerac strains exhibited a 7–15% reduction in mean survivorship relative to the N2 strain (Table 2 and Fig. 3, b and e). ANOVA analyses found a significant variance component for survivorship among the 4 strains (*F*_s_ = 16.8; *P < *0.0001) as well as among-line divergence (*F*_s_ = 1.42; *P < *0.05) (Table 3). As was observed for productivity, RW7000 exhibited the lowest survivorship among the 3 Bergerac strains relative to N2.

The Bergerac strains exhibited a 29–36% reduction in longevity relative to the N2 strain (Table 2 and Fig. 3, c and e). ANOVA analyses found a significant variance component for longevity among the 4 strains (*F*_s_′ = 12.49; *P < *0.0001) whereas among-line divergence was nonsignificant (Table 3).

The Bergerac strains exhibited delayed development to reproductive maturity relative to N2 (10–25% longer) (Table 2 and Fig. 3, d and e). ANOVA analyses found a significant variance component for developmental time among the 4 strains (*F*_s_′ = 34.06; *P < *0.0001) whereas among-line divergence was nonsignificant (Table 3). Relative to N2, the 3 Bergerac strains exhibited a 9–20% reduction in their developmental rate (Fig. 3e), with RW6999 and RW7000 exhibiting the greatest reduction in developmental rate.

All 3 Bergerac strains were significantly different from N2 for each of the 4 fitness traits (Fig. 3, a–e and Supplementary Tables 2–5). With respect to productivity, all pair-wise strain comparisons were significant with the relationship expressed as follows: RW7000 < CB4851 < RW6999 < N2 (Table 2 and Supplementary Table 2). With the exception of the CB4851 vs RW6999 comparison, all other pair-wise strain comparisons showed significant differences for survivorship to adulthood (RW7000 < RW6999, *P *=* *4.70_** × **_10^−3^; RW7000 < CB4851, *P *=* *1.90_** × **_10^−4^) (Table 2 and Supplementary Table 3). Survivorship to adulthood of the 4 strains showed the following trend: RW7000 < RW6999/CB4851 < N2. While all of the 3 Bergerac strains had significantly reduced mean longevity relative to N2, they did not differ significantly from each other with all the Bergerac strains surviving an average of 9–10 days after the L1 larval stage N2 (Table 2 and Supplementary Table 4). With the exception of the CB4851 vs RW7000 comparison, all other pair-wise strain comparisons showed significant differences for mean developmental time (RW6999 < RW7000, *P *=* *1.87_** × **_10^−6^; RW6999 < CB4851, *P *=* *5.94_** × **_10^−5^) (Table 2 and Supplementary Table 5). Developmental time of the 4 strains showed the following trend: N2 < RW6999 < RW7000 < CB4851/RW7000.

### Phenotypic divergence of Bergerac strains with respect to motility- and size-associated traits

The average speed and direction change of N2 worms was concordant with the values observed by Angstman *et al.* (2016), despite our use of a different worm tracking program. Our N2 average speed was 177.8 μm/s (Table 4) with an SD of 48.9 μm/s (SE 15.5) compared with an average 146.9 μm/s with an SD of 55.3 μm/s measured by Angstman *et al.* (2016). Similarly, our N2 average direction change (Table 4) was 0.531 radians/s with an SD of 0.218 radians/s (SE 0.069) compared with Angstman *et al.*’s (2016) average direction change of 0.70 radians/s with an SD of 0.30 radians/s, after converting their measurements from degrees to radians. Similarly, the average length of our N2 worms (799 μm, SD 56.2 μm, SE 17.8; Table 4) is close to a previously reported length of N2 after the fourth larval molt, 850 μm (Byerly *et al.* 1976).

**Table 4.** Motility- and size-associated traits of 3 *C. elegans* Bergerac strains relative to control worms of the laboratory strain, N2.

Caenorhabditis elegans strain	Speed (µm/s)	Length (µm)	Area (µm^2^)	Direction change (radians/s)
N2	178	799	61,078	0.53
CB4851	166	647	50,758	0.63
RW6999	121	675	49,966	0.59
RW7000	53	636	42,266	1.13

Traits measures for each of the 4 strains are for *n *=* *100 worms across 10 plates (10 worms/plate). Mean values for all 4 motility-related traits are provided for each of the 4 strains.

**Fig. 4.**
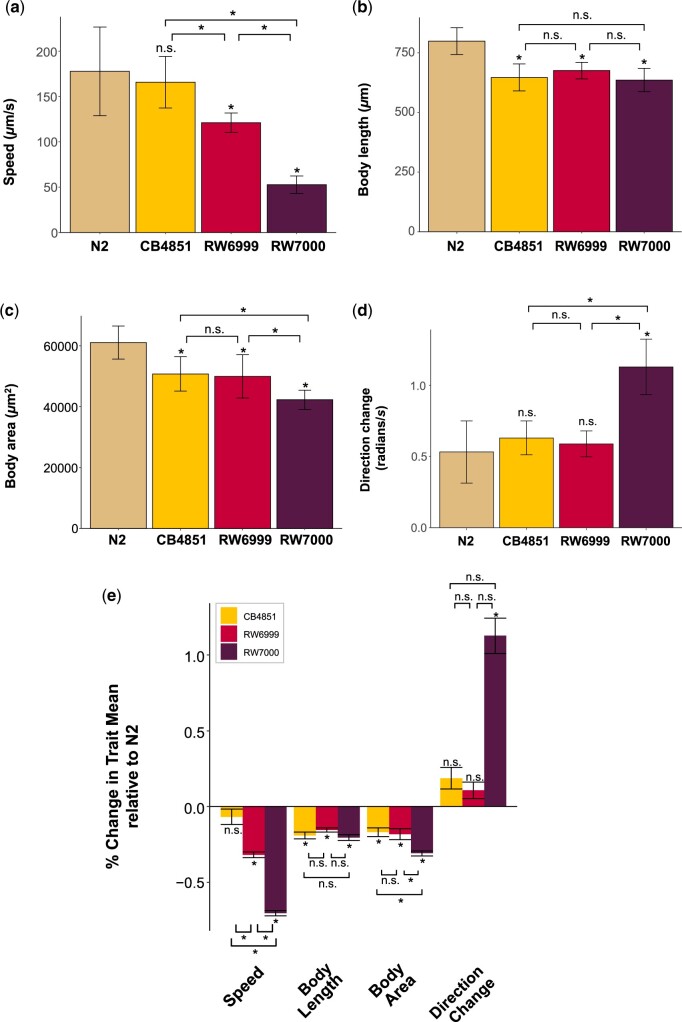
Physical size reduction and aberrant behavior of the Bergerac strains. Mean values for 4 traits measured in the motility analyses, namely a) speed, b) body length, c) body area, and direction change d) in the N2 control, and the 3 Bergerac strains CB4851, RW6999, and RW7000. e) Mean relative changes between the 3 Bergerac strains and the N2 control. For simplicity, the mean relative value for each of the 4 traits in the Bristol N2 control was scaled to a value of 1 (not displayed). The stars on brackets summarize *P*-values for Tukey–Kramer HSD comparisons between Bergerac strains. Stars on top of bars reflect *P-*values for Tukey–Kramer HSD comparisons to N2. Exact *P*-values for the pairwise strain comparisons using Tukey–Kramer HSD can be found in Supplementary Tables 6–9.

The Bergerac strains exhibited _**∼**_7–70% reduction in mean speed relative to the N2 strain (Table 4 and Fig. 4, a and e). ANOVA analyses found a significant variance component for speed among the 4 strains (*F*_s_ = 37.57; *P *=* *3.50_** × **_10^−11^) (Table 5). With the exception of the CB4851 vs N2 comparison, all other pair-wise strain comparisons showed significant differences for mean speed (RW7000 < CB4851, *P *=* *1.51_** × **_10^−9^; RW7000 < RW6999, *P *=* *4.14_** × **_10^−5^; RW6999 < CB4851, *P *=* *8.30_** × **_10^−3^) (Table 4 and Supplementary Table 6). Strain speed showed the following trend: N2/CB4851 > RW6999 > RW7000.

**Table 5.** ANOVA analyses for 4 motility and size-associated traits in the laboratory wild type strain N2 and 3 Bergerac strains (CB4851, RW6999, and RW7000) of *C. elegans*.

Source of variation	*df*	SS	MS	*F*_s_
Speed (µm/s)				
Among groups	3	96,109	32,036	37.57***
Within lines (error)	36	30,697	852	
Total	39	126,807		
Length (µm)				
Among groups	3	169,232	56,410	22.58****
Within lines (error)	36	89,950	2,498	
Total	39	259,182		

Area (µm^2^)				
Among groups	3	1,789,912,485	596,637,495	19.59****
Within lines (error)	36	1,096,184,461	30,449,568	
Total	39	2,886,096,946		

Direction change (radians/s)				
Among groups	3	2.288	0.763	28.15****
Within lines (error)	36	0.976	0.027	
Total	39	3.264		

* Significance level of 0.05.

** Significance level of 0.01.

*** Significance level of 0.001.

**** Significance level of 0.0001.

The Bergerac strains exhibited _**∼**_16–20% reduction in mean body length relative to the N2 strain (Table 4 and Fig. 4, b and e). ANOVA analyses found a significant variance component for body length among the 4 strains (*F*_s_ = 22.58; *P *=* *2.14_** × **_10^−8^) (Table 5). While all of the 3 Bergerac strains had significantly reduced mean body length relative to N2, they did not differ significantly from each other (Table 4 and Supplementary Table 7). Hence, body length showed the following trend: N2 > CB4851/RW6999/RW7000.

The Bergerac strains exhibited _**∼**_17–31% reduction in mean body area relative to the N2 strain (Table 4 and Fig. 4, c and e). ANOVA analyses found a significant variance component for body area among the 4 strains (*F*_s_ = 19.59; *P *=* *1.06_** × **_10^−7^) (Table 5). All 3 Bergerac strains had significantly reduced mean body area relative to N2 (Supplementary Table 8). The 3 Bergerac strains also exhibited some significant differences amongst each other with respect to body area. While the CB4851/RW6999 pair comparison was nonsignificant, RW7000 had significantly smaller body area relative to both CB4851 (*P *=* *7.73_** × **_10^−3^) and RW6999 (*P *=* *1.78_** × **_10^−2^) (Supplementary Table 8). Body area showed the following trend: N2 > CB4851/RW6999 > RW7000. Because all worms in these motility assays were approximately the same age, these size differences could be explained by the previously observed variation in developmental time. A Pearson test between the average developmental time for each strain and body area confirms that these variables are correlated (*P *=* *1.17_** × **_10^−6^).

The Bergerac strains exhibited _**∼**_11–113% increase in mean direction change relative to the N2 strain (Table 4 and Fig. 4, d and e). ANOVA analyses found a significant variance component for direction change among the 4 strains (*F*_s_ = 28.15; *P *=* *1.50_** × **_10^−9^) (Table 5). However, only RW7000 was significantly different from the 3 other strains (N2, and the other 2 Bergerac strains) with respect to direction change (Supplementary Table 9). The average direction change of strain RW7000 was much larger than the other Bergerac strains (RW6999 < RW7000, *P *=* *6.70_** × **_10^−8^; CB4851 < RW7000, *P *=* *3.72_** × **_10^−7^). The reason for the large difference in the average direction change displayed by RW7000 is also visually apparent. While wild-type worm locomotion occurs in an approximately sinusoidal trajectory, the low fitness RW7000 worms tend to lay still in a much straighter orientation than other strains with shallower wave magnitude during their sinusoidal locomotion. Because their ability to move forward and turn seems to be restricted, any changes in movement tend to be in a back-and-forth motion close to _**π**_ radians, while other strains tend to move forward and make slow turns. Direction change showed the following trend: N2/CB4851/RW6999 < RW7000.

### Local sequence context of Tc1 insertion sites

**Fig. 5.**
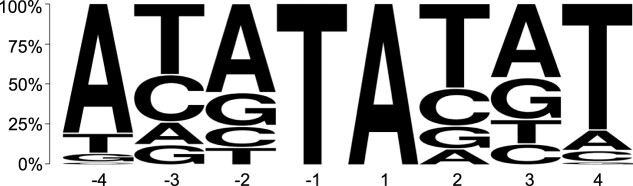
The Tc*1* insertion motif in the Bergerac strains. The vertical axis indicates the proportion of each base at 4 upstream and downstream positions from the insertion site. The motif for Tc*1* insertion sites was based on 1,659 insertions in this study and matched that presented in previous literature. Tc*1* invariably inserts between a T(−1) and an A(+1) with additional conserved A/T bases at positions −4 and +4.

Tc*1* had been well-documented to insert into 5_**′**_-TA-3_**′**_ target sites with a lightly conserved A/T rich motif (Rosenzweig *et al.* 1983b; Eide and Anderson 1988; Mori, Benian, *et al.* 1988). Korswagen *et al.* (1996) analyzed 83 Tc*1* insertion sites to conclude a symmetric consensus sequence for Tc*1* insertion, namely CAYA**TA**TRTG. We further analyzed 1,659 Tc*1* insertion sites in the 3 Bergerac strains to derive a consensus insertion motif (Fig. 5), confirming the conclusions of preceding studies (Eide and Anderson 1988; Korswagen *et al.* 1996). All the Tc*1* insertions were located at a 5_**′**_-TA-3_**′**_ target site within the consensus sequence. The consensus sequence was identified at 46,630 locations in the N2 reference genome (PRJNA13758.WS279).

### Nonrandom genomic distribution of Tc1 elements in the Bergerac strains

To determine whether Tc*1* proliferation in the Bergerac strains was random or influenced by genomic context, we classified the *C. elegans* genome based on 4 broad categories: (1) chromosomes, (2) recombination domains, (3) chromatin environment, and (4) exonic, intronic, or intergenic regions.

**Fig. 6.**
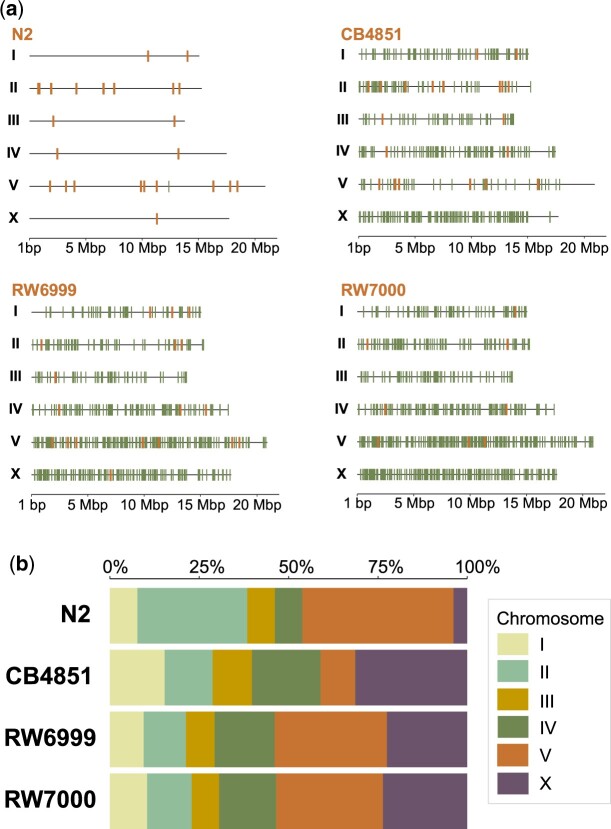
The chromosomal distribution of Tc*1* elements in the Bergerac strains relative to N2. a) Genomic map of Tc*1* insertions within each strain. Nonreference Tc*1* insertions are displayed in green, while reference Tc*1* sites in Bristol N2 are shown in orange. b) The proportion of Tc*1* elements by chromosome. Tc*1* elements were significantly overrepresented on the X chromosome in CB4851 (*χ*^2^ = 51.87, *P *=* *5.92 × 10^−13^), and chromosomes V and X in RW6999 (*χ*^2^ = 49.06, *P *=* *2.16 × 10^−9^) and RW7000 (*χ*^2^ = 50.88, *P *=* *9.15 × 10^−10^).

There is significant variation in chromosomal distribution of Tc*1* elements between the 3 Bergerac strains (*G *=* *96.27, *P *=* *3.33_** × **_10^−16^) (Fig. 6, a and b). This is primarily due to the relatively low number of Tc*1* elements on Chr. V in CB4851. When Chr. V was excluded from the analysis, the chromosomal distribution of Tc*1* was not significantly different between RW6999 and RW7000 (*G *=* *1.07, *P = *0.96), nor between the 3 Bergerac strains (*G *=* *4.32, *P = *0.83). Additionally, Tc*1* insertions were nonrandomly distributed across the 6 chromosomes (5 autosomes and X) in the 3 Bergerac strains once corrected for chromosome length. Tc*1* elements were overrepresented on Chr. V and X in the closely related strains RW6999 (*χ*^2^ = 49.06, *P *=* *2.16_** × **_10^−9^) and RW7000 *(χ*^2^ = 50.88, *P *=* *9.15_** × **_10^−10^). This Tc*1* overrepresentation was particularly pronounced on chromosome V which had 48% and 40% more Tc*1* elements than expected based on chromosome length alone in strains RW6999 and RW7000, respectively. When the number of 8 bp consensus Tc*1* insertion sites *(*5_**′**_-AYATATRT-3_**′**_) per chromosome is taken into account, the number of Tc*1* are 56% and 49% greater on chromosome V than expected in RW6999 and RW7000, respectively. Tc*1* number was better correlated with chromosome size (*R*^2^ = 0.96 for RW6999) than the number of consensus Tc*1* insertion sites per chromosome (*R*^2^ = 0.70 for RW6999).

**Fig. 7.**
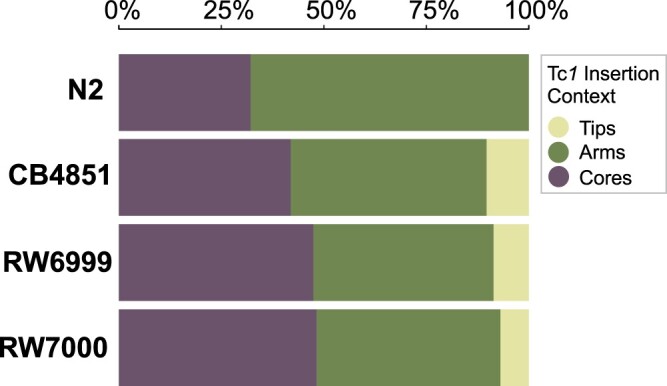
The proportions of Tc*1* elements located in arms, cores, and tips. Arms are associated with high recombination rates whereas cores and tips have low recombination rates. There were no significant differences in the distribution of Tc*1* between these 3 domains (N2: *χ*^2^ = 2.66, *P = *0.21; CB4851: *χ*^2^ = 0.47, *P = *0.49; RW6999: *χ*^2^ = 4.39, *P = *0.11; RW7000: *χ*^2^ = 4.81, *P = *0.11).

The genome was additionally split into arms, cores, and tips based on recombination domains designated by Rockman and Kruglyak (2009) (Fig. 7). Gene-poor high-recombination arms, gene-rich low-recombination cores, and gene-poor low-recombination tips comprise 45.7%, 47%, and 7.3% of the *C. elegans* genome, respectively. The distribution of Tc*1* insertions in arms and cores was not significantly different from random expectation within N2 (*χ*^2^ = 2.66, *P = *0.21), CB4851 (*χ*^2^ = 0.47, *P = *0.49), RW6999 (*χ*^2^ = 4.39, *P = *0.11), and RW7000 (*χ*^2^ = 4.81, *P = *0.11) corrected for multiple-comparisons (Holm–Bonferroni method; Holm 1979). Furthermore, there was no significant difference between cores and arms in Tc*1* insertions that are shared between Bergerac strains and insertions that are unique to a particular strain (Supplementary Fig. 2; *G *=* *3.24, *P = *0.072). Additional analysis of chromosomal tips was excluded because they represent such a small proportion of the genome.

**Fig. 8.**
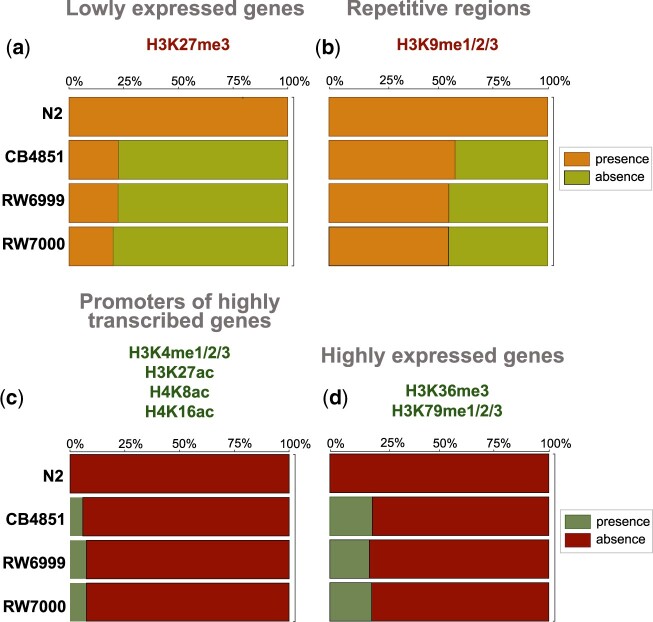
The proportions of Tc*1* insertions across different chromatin domains. a) Histone modification H3K27me3 is associated with repressed chromatin and lowly expressed genes. All of the Tc*1* positions in N2 were found in this domain. Tc*1* insertions were more abundant in regions associated with H3K27me3 than expected by chance in CB4851 (*χ*^2^ = 7.44, *P = *0.013) and RW6999 (*χ*^2^ = 8.82, *P = *0.0089), but not RW7000 (*χ*^2^ = 2.82, *P = *0.09). b) Histone modifications H3K9me1/2/3 are associated with repetitive DNA. All of the Tc*1* positions in N2 were found in these domains. Tc*1* insertions were overrepresented in H3K9me1/2/3 domains in strains RW6999 (*χ*^2^ = 5.06, *P = *0.049) and RW7000 (*χ*^2^ = 7.06, *P = *0.024), but not in CB4851 (*χ*^2^ = 7.06, *P = *0.44). c) Histone modifications H3K4me1/2/3, H3K27ac, H4K8ac, and H4K16ac are associated with promoters of highly expressed genes. In N2, Tc*1* insertions were entirely absent from these domains. Tc*1* insertions were underrepresented in domains associated with H3K4me1/2/3, H3K27ac, H4K8ac, H4K16ac, and promoters of in all 3 Bergerac strains: CB4851 (*χ*^2^ = 10.91, *P = *0.00287), RW6999 (*χ*^2^ = 5.96, *P = *0.0184), and RW7000 (*χ*^2^ = 6.79, *P = *0.0184). d) Histone modifications H3K36me3 and H3K79me1/2/3 are associated with highly expressed genes. In N2, Tc*1* insertions were entirely absent from these domains. In all 3 Bergerac strains, Tc*1* insertions were underrepresented in these domains (CB4851: *χ*^2^ = 48.33, *P *=* *3.60 × 10^−12^; RW6999: *χ*^2^ = 75.28, *P *=* *8.17 × 10^−18^; RW7000: *χ*^2^ = 82.71, *P *=* *2.85 × 10^−19^).

To ascertain transposition into different chromatin environments, 5 previously identified broad patterns of histone modification (Liu *et al.* 2011) were used to gate Tc*1* insertions in the 3 Bergerac strains relative to the Bristol N2 strain. These 5 patterns of histone modification are as follows: (1) lowly expressed genes (H3K27me3), (2) repetitive regions (H3K9me1/2/3), (3) dosage compensation (H3K27me1, H4K20me1), (4) promoters of highly expressed genes (H3K4me1/2/3, H3K27ac, H4K8ac, and H4K16ac), and (5) highly expressed genes (H3K36me3 and H3K79me1/2/3). As there was potential overlap between histone modification context, each category was tested individually for each strain. Interestingly, all Tc*1* locations identified in N2, both in the reference genome and in our laboratory isolate, inhabited regions associated with H3K27me3 or H3K9me1/2/3, and canonically identified as repressed in the *C. elegans* genome (Fig. 8, a and b), while being absent from regions associated with high expression (Fig. 8, c and d).

No significant preferences were found in Tc*1* insertions in domains associated with dosage compensation and enriched on the X chromosome (H3K27me1, H4K20me1) in any of the Bergerac strains (CB4851: *χ*^2^ = 0.003, *P *=* *1; RW6999: *χ*^2^ = 2.12, *P = *0.40; RW7000: *χ*^2^ = 0.44, *P *=* *1, Holm-Bonferroni corrected) (not shown in Fig. 8). There were no significant differences between the 3 Bergerac strains with regards to Tc*1* insertions in any of the 5 chromatin modification regions analyzed here (4 shown in Fig. 8). In heterochromatic regions associated with H3K27me3 and low gene expression (Fig. 8a), Tc*1* insertions were more abundant than expected by chance in CB4851 (*χ*^2^ = 7.44, *P = *0.013) and RW6999 (*χ*^2^ = 8.82, *P = *0.0089), but not RW7000 (*χ*^2^ = 2.82, *P = *0.09) after correcting for multiple comparisons (Holm 1979). Chromatin regions associated with H3K9me1/2/3, repetitive DNA and low gene expression (Fig. 8b), were also slightly enriched for Tc*1* insertions in RW6999 (*χ*^2^ = 5.06, *P = *0.049) and RW7000 (*χ*^2^ = 7.06, *P = *0.024), but not in CB4851 (*χ*^2^ = 7.06, *P = *0.44). Domains associated with H3K36me3, H3K79me1/2/3 and high gene expression (Fig. 8d) had fewer Tc*1* insertions than expected by chance in all 3 Bergerac strains (CB4851: *χ*^2^ = 48.33, *P *=* *3.60_** × **_10^−12^; RW6999: *χ*^2^ = 75.28, *P *=* *8.17_** × **_10^−18^; RW7000: *χ*^2^ = 82.71, *P *=* *2.85_** × **_10^−19^, Holm-Bonferroni corrected). Similarly, domains associated with promoters of highly expressed genes (H3K4me1/2/3, H3K27ac, H4K8ac, H4K16ac, Fig. 8c) had fewer Tc*1* insertions than expected by chance (CB4851: *χ*^2^ = 10.91, *P = *0.00287; RW6999: *χ*^2^ = 5.96, *P = *0.0184; RW7000: *χ*^2^ = 6.79, *P = *0.0184, Holm-Bonferroni corrected). Some of the Tc*1* insertions associated with highly expressed genes in the Bergerac strains were found in germline enriched genes (Han *et al.* 2019). CB4851, RW6999, and RW7000 harbored 10, 17, and 21 Tc*1* insertions within or near highly expressed germline enriched genes, respectively. In sum, repressed chromatin had excess insertions and transcriptionally active chromatin had fewer Tc*1* insertions than expected by chance. However, Tc*1* insertions are significantly more likely in transcriptionally active chromatin in the Bergerac strains than in N2 (Fisher’s exact test: N2 vs CB4851, *P*_adj_ = 0.0243; N2 vs RW6999, *P*_adj_ = 0.0272; N2 vs RW7000, *P*_adj_ = 0.0243).

**Fig. 9.**
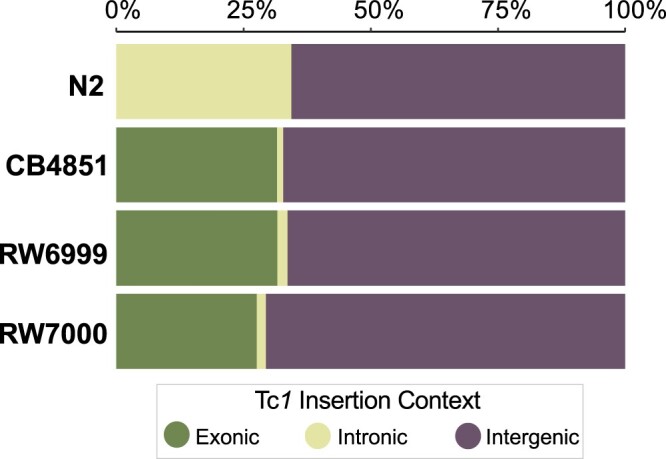
The proportion of Tc*1* elements in exons, introns, and intergenic regions of Bergerac strains in comparison to Bristol N2. In N2, the Tc*1* insertions were only found in introns and intergenic regions. The proportion of Tc*1* insertions in exons, introns, and intergenic regions was significantly different between Bristol N2 and the Bergerac strains (*G *=* *54.26, *P *=* *6.54 × 10^−10^, *df *=* *6), with the Bergerac strains showing a far larger and fewer proportion of Tc*1* insertions in exonic regions and intronic regions, respectively. There was no significant difference among the 3 Bergerac strains in the proportion of Tc*1* insertions in exons, introns, and intergenic regions (*G *=* *4.28, *P = *0.37, *df *=* *4).

There were significant differences between the 4 strains in the distribution of Tc*1* elements in exons, introns, and intergenic regions (*G *=* *54.26, *P *=* *6.5_** × **_10^−10^) (Fig. 9). None of the Tc*1* insertions in N2 were present in exons; rather they appear to be overrepresented in introns (Fischer’s exact: *P *=* *1.3_** × **_10^−5^). The distribution of Tc*1* is not significantly different across exons, introns, and intergenic regions between the 3 Bergerac strains (*G *=* *4.28, *P = *0.28). However, Tc*1* appears to be underrepresented in exons and overrepresented in introns and intergenic regions in RW6999 (*χ*^2^ = 10.20, *P = *0.012, adjusted for multiple comparison) and RW7000 (*χ*^2^ = 22.32, *P *=* *5.6_** × **_10^−5^, *P* adjusted by Holm-Bonferroni correction). The underrepresentation of Tc*1* in exons could be due to selection. However, potential Tc*1* insertion sites are also more abundant in introns and intergenic regions due in part to their higher A/T content compared with exons. If we recalculate the expected number of Tc*1* insertions in exons, introns and intergenic regions based on the frequency of the consensus integration sequence in these categories, Tc*1* insertions are overrepresented in exons and underrepresented in intergenic regions (CB4851: *χ*^2^ = 38.61, *P *=* *8.27_** × **_10^−9^; RW6999: *χ*^2^ = 59.53, *P *=* *3.55_** × **_10^−13^; RW7000: *χ*^2^ = 35.14, *P *=* *2.35_** × **_10^−8^, *P* adjusted by Holm-Bonferroni correction). For example, in Bergerac strain RW6999, the expected number of Tc*1* insertions in exons is 121 based on the frequency of consensus insertions sites whereas the observed number is 190.

### Mutations in the Bergerac strains

*Tc1* insertions were found to disrupt the exons of many protein-coding genes. Since the insertion of TEs into exons can lead to a disruption or total knock-out of gene function (Orgel and Crick 1980; Muñoz-López and García-Pérez 2010), the RNAi phenotypes for these genes provides a prediction for the effect of these insertions. These RNAi phenotypes (Supplementary Table 10) include many phenotypes observed in the phenotypic assays, including reduced brood size, slow growth, embryonic lethality, shortened life span, and locomotion defects. In addition, the Bergerac strains share 6,191 homozygous SNPs/indels after filtering (see *Materials and methods*). These mutations were predicted to result in an amino acid change in 384 genes (Supplementary Table 11) and 122 of these mutations were predicted to have a deleterious effect on protein function by SIFT (Vaser *et al.* 2016). No candidate mutations were identified in genes known to be relevant for the small RNA pathways linked to regulation of TE activity (Supplementary Table 11; Billi *et al.* 2014). However, the protein *abt-1* was annotated by WormBase as being involved in germline RNAi. Despite a lack of damage to sRNA-related genes, several genes related to meiosis, and recombination (*him-8*, *msh-5*, *rec-8*, *zyg-12*, and *dsb-1*) were predicted to have deleterious amino acid changes by SIFT (Vaser *et al.* 2016). The MiModd package also produced calls for deletions in the Bergerac strains. However, no exonic deletions in genes potentially involved in TE suppression were identified.

### Dating the divergence of Bergerac and N2 (Bristol) strains of C. elegans

In order to generate divergence time estimates among the Bergerac strains and the N2 (Bristol) strain, we employed whole-genome sequence (WGS) data to determine mtDNA genetic divergence (SNPs) between N2 and each of the 3 focal Bergerac strains. N2–RW7000, N2_**−**_RW6999, and N2_**−**_CB4851 differed by 4 SNPs each. We assumed (1) a spontaneous base substitution rate of 4.32_** × **_10^−8^/site/generation for the *C. elegans* mtDNA genome (Konrad *et al.* 2017) and (2) 3 estimates of generation time (14-, 30- and 60-day generation) in wild *C. elegans* which is more apt based on the ecology of the species (Cutter 2008). Assuming a generation time of 14, 30, and 60 days, wild *C. elegans* are expected to have approximately 26, 12, and 6 generations per year, respectively. Under this range of generation time, all 3 Bergerac strains are estimated to have diverged from N2 between 129 and 552 years (14-day generation: 129 years; 30-day generation: 276 years; 60-day generation: 552 years). Finally, to place the Bergerac strains in a phylogenetic context, we ran a maximum likelihood analysis (PhyML) on 11 strains, which included the 3 Bergerac strains and N2 using both mtDNA and nuclear SNPs. The phylogenetic tree matched the expected relationships, with the French natural isolates JU394 and JU2565 displaying the closest relationship to the Bergerac strains, and the Bergerac strains forming a monophyletic group on the tree (Supplementary Fig. 2) (Cook *et al.* 2017).

## Discussion

The Bergerac and the N2 (Bristol) strains were the first 2 laboratory isolates of *C. elegans* and hence, of historical importance in the development of the species as a model organism (Riddle *et al.* 1997). Laboratory culturing of the Bergerac strain preceded that of N2 by several decades (Nigon 1949; Nigon and Dougherty 1949). However, the Bergerac strain displayed temperature-sensitivity (Fatt and Dougherty 1963), low fertility (Abdulkader and Brun 1980; Lee *et al.* 2016), and other phenotypic abnormalities including uncoordinated movement (Emmons *et al.* 1983; Hodgkin and Doniach 1997), which ultimately led to its supplantation in the laboratory by the more phenotypically robust Bristol N2 strain (Brenner 1974; Sulston and Brenner 1974). A series of well-executed studies in succession over a decade from the late 1970s to late 1980s served to elucidate the genetic bases of some of these phenotypic peculiarities that have since become characteristic of the original Bergerac isolate and its descendants (see *Introduction*), namely the proliferation of a unique DNA transposon called Tc*1* (Emmons *et al.* 1979, 1983; Liao *et al.* 1983; Eide and Anderson 1985; Egilmez *et al.* 1995). Sequencing of a Tc*1* element showed it to be 1,610 bp in length (coding potential of 343 aa) with short inverted terminal repeats of 54 bp (Rosenzweig *et al.* 1983a). Interestingly, all copies of Tc*1* in *C. elegans* appeared to be uniform in length and largely conserved in their DNA sequence, suggesting that Tc*1* coded for products mediating its own transcription (Liao *et al.* 1983) or that the regulatory mechanism for transposition required a full-length element (Mori, Moerman, *et al.* 1988). The transposition of Tc*1* was shown to be strain-specific (Eide and Anderson 1985) with the Bergerac strain BO (RW7000) capable of generating spontaneous mutations in the *unc-22* locus at a per generation rate of approximately 10^−4^, and 3 orders of magnitude higher than the baseline spontaneous mutation rate at the same locus in the N2 genetic background (Moerman and Waterston 1984). Furthermore, while both the Bergerac (RW7000 subclone) and N2 strains exhibited high frequencies of somatic excision (Emmons and Yesner 1984; Emmons *et al.* 1986), the Bergerac strain was unique in having active germline transposition (Collins *et al.* 1987). It should be mentioned that the history and nomenclature of the Bergerac strains can be confusing given that clones were shared with multiple laboratories over a period of decades and sublineages were often given new names (reviewed in Nigon and Félix 2017). While RW6999 is a subclone of Bergerac RW7000/BO/LY, the Bergerac strain CB4851 has high Tc*1* content (Hodgkin and Doniach 1997) but has been reported to lack the active germline transposition of Tc*1* (Moerman *et al.* 1986; Nigon and Félix 2017) as observed in the RW7000 and RW6999 strains (Moerman and Waterston 1984; Eide and Anderson 1985). Despite a decade of sustained focus in the pregenomics era, a modern genomic analysis of the genomic distribution of TEs, a precise quantification of their relative fitness, and further investigations into a possible cause for TE deregulation in the Bergerac strains is hitherto lacking.

The TE Tc*1* was shown to be unique to *C. elegans* and while present in low copy-number in the majority of *C. elegans* strains examined, the Bergerac strain and its descendants were classified as high copy-number strains. The standard laboratory strain of *C. elegans*, Bristol N2, has diverged genetically since it was first isolated through the independent accumulation of base substitutions, copy-number changes and TE activity during propagation in different laboratories (Lipinski *et al.* 2011). The estimated number of Tc*1* elements in low Tc*1* copy-number strains, such as Bristol N2 typically ranges from 20 to 30 copies (Emmons *et al.* 1983; Liao *et al.* 1983; Egilmez *et al.* 1995). Emmons *et al.* (1983) were the first to use Southern blotting to estimate genomic Tc*1* copy number in the N2 and Bergerac strain RW7000 (also referred to as the BO subclone) as 20_** ± **_5 and 200_** ± **_50, respectively. Liao *et al.* (1983) estimated Tc*1* copy-number in the N2 and Bergerac strain RW7000 (also referred to as the LY subclone) to be 25–30 and several hundred copies, respectively. Eide and Anderson (1985) mention Bergerac RW7000/BO/LY lineage as possessing 250 Tc*1* copies as per Emmons *et al.* (1983) and Liao *et al.* (1983). Mori, Moerman, *et al.* (1988) subsequently found the RW7000/BO/LY lineage to harbor >300 Tc*1* copies. Hence, Southern blotting techniques established a range of 25–30 and 200 to >300 Tc*1* copies for the N2 and RW7000 strain, respectively. Recognizing the limitation of Southern blots in the accurate estimation of Tc*1* in high-copy strains, Egilmez *et al.* (1995) opted to use DNA dot-blots. N2 and Bergerac RW7000/BO/LY were estimated to possess 31 and 515_** ± **_48 Tc*1* copies, respectively (Egilmez *et al.* 1995). More recently, Laricchia *et al.* (2017) were the first to estimate transposon abundance in several hundred *C. elegans* isolates from Illumina pair-end sequencing data. One Bergerac sublineage, CB4851, was included in their study and estimated to possess 406 Tc*1* copies (Laricchia *et al.* 2017). However, because there are no existing previous hybridization-based estimates of Tc*1* count for CB4851, it is not possible to compare transposon number estimates generated from hybridization vs WGS data. This study offers new estimates of Tc*1* counts for 3 Bergerac strains based on both (1) bioinformatic analyses of WGS data and (2) independent confirmation by ddPCR. We determined far higher copy-number estimates of Tc*1* in the Bergerac strains than preceding studies with median values of 451, 581, and 748 copies for CB4851, RW6999, and RW7000, respectively. In the best-studied RW7000 Bergerac strain, our estimate of Tc*1* copy-number is more than 2_**×**_ greater than past studies relying on Southern hybridizations (Emmons *et al.* 1983; Liao *et al.* 1983; Mori, Moerman, *et al.* 1988) and approximately 1.5_**×**_ greater than an estimate based on DNA dot-blots (Egilmez *et al.* 1995). The discrepancy in our estimates of Tc*1* copy-number and those of past studies relying on Southern hybridizations and DNA dot-blots is likely due to drawbacks associated with the latter techniques, namely membrane saturation limiting resolution of copy-number when counts exceed _**∼**_100. The 5 bioinformatic methods employed to estimate TE copy-number were consistent with each other, both in the number of calls per strain and in the differences in Tc*1* copy-number between the strains. Furthermore, 90% and 70% of the calls were shared by more than one method and by all methods, respectively. Nevertheless, these median numbers of Tc*1* are likely to be underestimates as both sequence read coverage and ddPCR yielded even higher estimates of Tc*1* copy-number in the 3 Bergerac strains.

Both our ddPCR and bioinformatic approaches demonstrate that Tc*1* copy-number can vary considerably among the 3 Bergerac sublineages/strains examined in this study (median values of 451, 581, and 748 copies for CB4851, RW6999, and RW7000, respectively). This among-strain variation in Tc*1* copy-number could be owing to either one or a combination of at least 2 factors. First, it is possible that some Bergerac sublineages have evolved a mechanism of TE regulation that limits further proliferation of Tc*1*. For example, CB4851 is thought to lack the active germline transposition characteristic of the RW7000 and RW6999 strains (Nigon and Félix 2017). Second, the complete history of laboratory propagation for these strains over multiple decades is obscure, with some strains possibly having been subjected to lengthier periods of cryopreservation or laboratory evolution than others.

Aside from Tc*1*, the only consistent difference in TE copy-number between the Bergerac strains and N2 was for Tc*2*. The average number of Tc*2* elements was 20 and 3 in the Bergerac strains and N2, respectively, and normalized read depth suggests 25–30 copies of Tc*2* in the Bergerac strains and 4 in N2. Tc*2*, originally discovered in a Bergerac strain, was upon its discovery found in higher copy-number Tc*1* strains than in low-copy number Tc*1* strains (Levitt and Emmons 1989). This coincidence suggested that the proliferation of Tc*1* and Tc*2* was due to shared copy-number regulation mechanisms (Levitt and Emmons 1989). Since then, many genes have been discovered in *C. elegans* that influence the transposition of multiple TEs (Vastenhouw *et al.* 2003; Lee *et al.* 2012; Weick and Miska 2014; McMurchy *et al.* 2017; Wallis *et al.* 2019). In contrast to the striking differences among the Bergerac strains with respect to the number of Tc*1* insertions, Tc*2* copy-number and locations are remarkably well-conserved. It appears that the explosive proliferation and divergence of Tc*1* insertions in the Bergerac strains is caused by factors specific to Tc*1* and not shared with other TEs in *C. elegans.*

While Bergerac strains have long been observed to harbor mutational defects (Wood *et al.* 1980; Emmons *et al.* 1983), this study is the first to offer precise quantification of their low composite fitness via phenotypic assays of 4 fitness-related traits (productivity, survivorship to adulthood, developmental rate, and longevity). Notably, all 3 Bergerac strains differ significantly from N2 with respect to each of the 4 fitness traits; in each case, their mean fitness trait values are far lower than N2. However, it should be noted that the fitness values reported here for the 2 active mutator strains (RW7000 and RW6999) may vary among experiments owing to their dynamic genomes. Productivity and survivorship to adulthood are 2 traits considered most germane to organismal fitness. The 3 Bergerac strains exhibited an extensive reduction (65–86%) in productivity relative to the N2 strain. To place this in perspective, spontaneous mutation accumulation (MA) lines of *C. elegans* subjected to extreme genetic drift and inbreeding for >400 consecutive generations displayed an average 44–55% reduction in productivity (Katju *et al.* 2015, 2018). Mean survivorship to adulthood in the 3 Bergerac strains was reduced by 7–15% relative to N2. In contrast to productivity, this reduction in survivorship of the Bergerac strains is on par with the 12–19% decline observed in the same long-term spontaneous MA lines of *C. elegans* evolved over 400 generations (Katju *et al.* 2015, 2018). The Bergerac strains also exhibited reduced longevity (29–36% lower relative to N2) as well as delayed developmental rate (9–20% longer than N2). These experiments do not bear on the question whether TE proliferation alone directly contributes to all observable fitness decline in the Bergerac strains. For example, a temperature-sensitive point mutation in the *zyg-12* gene in *C. elegans* renders it almost completely sterile at 25_**°**_C (Malone *et al.* 2003). The presence of this mutant *zyg-12* allele was confirmed in all 3 Bergerac strains in this study. Our study corroborates previous findings that the temperature-sensitivity of Bergerac is not owing to Tc*1* transposition, but due to an independent point mutation in *zyg-12* (Nigon and Félix 2017). However, TE mobilization events have been shown to have a direct negative impact on host fitness; notable examples include the reduction of host fertility and viability following *P*-element invasion in *Drosophila* (Kidwell and Novy 1979), decreased fitness and egg hatchability in laboratory lines of *D. melanogaster* with elevated TE transposition rates (Pasyukova *et al.* 2004), and fitness loss in *Drosophila* MA lines following *copia* insertions (Houle and Nuzhdin 2004). It is therefore reasonable to assume that the low fitness of the Bergerac strain and its derivatives is owing largely to the unchecked proliferation of TEs.

In addition to confirming that all Bergerac strains had significantly lower fitness than N2, our study quantified significant interstrain differences among the 3 Bergerac strains studied here with respect to fitness. RW7000 (or Bergerac-BO), used in many transposon-tagging studies in the 1990s (Korswagen *et al.* 1996), exhibited the lowest means for 3 traits (productivity, survivorship, and longevity). RW7000 also possesses the highest Tc*1* copy-number among the 3 strains studied here. Interestingly, the strain RW6999, listed as an RW subclone of RW7000 by the *Caenorhabditis* Genetics Center (CGC), displayed the highest fitness among the Bergerac strains, despite having the second-highest number of Tc*1* insertions. The hypothesis that TE proliferation is the sole cause of fitness differences between the 3 Bergerac strains is ruled out by these results. It appears that fitness is not simply correlated with copy-number of active Tc*1* elements but other determinants as well such as site(s) of genomic integration, and/or certain key loci with high mutator activity.

Bergerac strains are also known to display abnormal phenotypes with respect to locomotion, and are often referred to as “uncoordinated” (Brenner 1974; Hodgkin and Doniach 1997; Emmons *et al.* 1983; Nigon and Félix 2017). The *unc-22* gene on Chr. IV plays an important role in muscle structure and function (Moerman *et al.* 1988) and is a frequent target for Tc*1* element insertions in the Bergerac strains (Moerman *et al.* 1986). As was the case with fitness-related traits, all 3 Bergerac strains had significantly reduced mean body length and body area relative to N2. In addition, some Bergerac strains exhibit significant deviations from N2 with respect to locomotion. RW6999 and RW7000 have significant reductions in speed relative to N2. In addition, RW7000 worms displayed a compromised ability to move forward and turn, resulting in a significant increase in direction change relative to N2. Hence, it appears that RW7000 worms have the most severe impairment in locomotion relative to N2. It is likely that Tc*1* insertions have contributed to the observed changes in these quantitative traits in conjunction with other independent mutations in the unique evolutionary trajectory of the Bergerac ancestor and its derived lineages. One mutation present in all 3 Bergerac strains was a nonsynonymous base substitution in an olfactory G-protein-coupled receptor *str-208* yielding a Cys226Ser/Tyr. This mutation may result in improper folding of the protein, with possible inhibitory effects on proper signaling and sensation of external stimuli. The inability to properly relay/convey external stimuli could explain the erratic movement exhibited by the Bergerac strains in the motility assays. The Bergerac strains were additionally noted to leave the area of the bacterial feeding lawn or position themselves around the bacterial seed instead of entering to feed. This behavior could be rooted in a dysfunctional olfactory system and possibly contribute to diminished fitness.

The genomes of multicellular eukaryotes are an assemblage of DNA sequences necessary for function as well as repetitive DNA sequences that may not contribute to organismal function. This latter component, which may constitute a large fraction of eukaryotic genomes, includes TEs that can be detrimental to the host genome when they transpose into coding regions thereby causing gene disruption. The genome has therefore been likened to an ecological community with TEs being functionally regarded as parasitic invaders with the host genome under selective pressure to actively engage in tackling this TE spread (Brookfield 2005; Bourque *et al.* 2018). Hence, the distribution and abundance of TEs across the genome is frequently nonrandom due to a combination of natural selection, as well as TE integration site preferences, and deletions (Bourque *et al.* 2018). TEs are a source of both germline and somatic mutations, contribute to ectopic recombination and can interfere with normal gene expression, all of which are, on average, deleterious to host fitness. Consequently, TEs are observed less frequently within coding and regulatory DNA sequences than expected by chance. Furthermore, the distribution of TEs in genomes can also depend on the local recombination rate due to the effects of natural selection against both ectopic exchange and insertional mutations. For example, the numbers of DNA-based TEs are negatively correlated with recombination rate in the *Drosophila melanogaster* genome (Rizzon *et al.* 2002). Assuming that opportunities for ectopic recombination between TEs increase with the local recombination frequency, the presence of TEs is more likely to be detrimental in regions with high recombination rates compared with low recombination rates (Langley *et al.* 1988). Moreover, natural selection is less efficient in regions with low recombination relative to high recombination, which predicts that deleterious TE insertions are more likely to accumulate in regions of low recombination (Hill and Robertson, 1966; Dolgin and Charlesworth 2008). In addition, insertional preferences may vary with nonrandom distribution of features such as suitable integration motifs, recombination rate, and chromatin organization (Sultana *et al.* 2017). Tc*1* transpositions in *C. elegans* have been shown to occur more frequently both (1) within than between chromosomes and (2) to proximate than distant locations on the same chromosome (Fischer *et al.* 2003). Consequently, this bias toward proximate locations violates the usual assumption of independence in statistical tests. If, for now, we ignore these limitations to interpreting the genome-wide distribution of Tc*1* elements, we find that Tc*1* is overrepresented on Chr. V in strains RW6999 and RW7000, and on the X chromosome in all 3 Bergerac strains after correcting for chromosome length. Interestingly, gene copy-number changes appear to be more common on Chr. V relative to the rest of the genome. Although Chr. V represents 21% of the *C. elegans* genome, it incurred >40% of the spontaneous copy-number changes in experimental *C. elegans* lines (Konrad *et al.* 2018). Moreover, Chr. V also appears to be enriched for CNVs in natural populations (Maydan *et al.* 2007, 2010; Thompson *et al.* 2013). While we lack a mechanistic explanation for this enrichment of Tc*1* on Chr. V in the Bergerac strains, we speculate it may be causally related to the propensity for CNVs on Chr. V. In *C. elegans*, the chromosome arms are relatively gene-poor regions but have higher recombination frequency than chromosome cores, which are also gene-rich (Rockman and Kruglyak, 2009). In contrast to the relationship between recombination rate and TEs in *Drosophila*, TE abundance in *C. elegans* is greater in the high-recombination arms than in the low-recombination cores (Duret *et al.* 2000; Laricchia *et al.* 2017). We did not find a significant difference in the abundance of Tc*1* elements between the chromosome arms and cores across the 3 Bergerac strains analyzed here. In this regard, our results are similar to an earlier analysis of TEs in *C. elegans* that also failed to find a significant relationship between recombination rate and Tc*1* copy-number (Duret *et al.* 2000).

Tc*1* is underrepresented in exons which is consistent with selection against strongly deleterious insertions in coding regions of the genome. However, the consensus Tc*1* integration motif is more commonly found in introns and intergenic regions. The underrepresentation of Tc*1* abundance in exons could therefore primarily be a function of the availability of suitable integration motifs. In a similar vein, Tc*1* insertions in the Bergerac strains were slightly less and more abundant than expected by chance in transcriptionally active and repressed chromatin, respectively. This pattern could arise by insertional preference into repressed chromatin, unequal distribution of integration motifs between repressed and transcriptionally active chromatin, or selection against insertions into transcriptionally active chromatin. Indeed, there is strong purifying selection on TE expression in experimental populations of *C. elegans* (Bergthorsson *et al.* 2020). Furthermore, there is a striking difference between the Bristol N2 and the Bergerac strains in that the former sequenced for this study retain Tc*1* insertions exclusively in repressive chromatin. The Bergerac strains contained none of the alleles that have previously been found to derepress Tc*1* activity (Vastenhouw *et al.* 2003; Lee *et al.* 2012; Weick and Miska 2014; McMurchy *et al.* 2017; Wallis *et al.* 2019) and mapping putative mutator loci for Tc*1* in these strains suggested that the mutator activity was associated with multiple sites in the genome that were themselves mobile (Moerman and Waterston 1984; Mori, Moerman, *et al.* 1988b). The presence of numerous Tc*1* elements insertions in transcriptionally active chromatin in the Bergerac strains could explain their high rate of germline transposition. Even if Tc*1* preferentially integrates into repressed chromatin, thus evading purifying selection to some degree, serendipitous insertions of Tc*1* into an active chromatin environment could create a proliferative loop of Tc*1* expansion that selection is simply unable to completely restrain.

The evolutionary history of the Bergerac strain and its derivatives remains obscure. The current Bergerac strain was isolated by Victor Nigon from his garden soil in Bergerac, France in 1944 (Nigon and Félix 2017). How evolutionary diverged are the Bergerac and the N2 strains? Both earlier and recent studies of *C. elegans* intraspecific genetic diversity have similarly observed that single-nucleotide variant (SNV) diversity is often shared among many natural isolates and that the species is characterized by lower levels of genetic diversity relative to other obligately outcrossing as well as facultatively selfing species in the genus *Caenorhabditis* (Denver *et al.* 2003; Sivasundar and Hey 2003; Cutter 2006; Dey *et al.* 2013; Thomas *et al.* 2015). Moreover, modern approaches to ascertain species-wide diversity with genome-wide coverage have confirmed this paucity of genetic diversity, with evolutionarily recent chromosome-scale selective sweeps being implicated as one of the major causes for this pattern (Andersen *et al.* 2012; Crombie *et al.* 2019; Lee *et al.* 2021). Based on mtDNA genetic divergence among the 3 Bergerac strains and N2 and assuming 3 different estimates of generation time in the wild, we estimated a very evolutionary recent N2-Bergerac divergence range of 129–552 years. As expected, the 3 focal Bergerac strains in this study form a monophyletic clade and are more closely related to 2 French natural isolates, providing evidence of some biogeographic structure. Another pertinent outstanding question is whether the high activity of Tc*1* transposition in the Bergerac germline originated in the wild or occurred following laboratory domestication. Interestingly, the subclones/derivatives of Bergerac display variation with respect to active germline transposition of Tc*1* (Emmons *et al.* 1983; Liao *et al.* 1983; Moerman and Waterston 1984). For example, the BO/RW7000 sublineage has active germline transposition (Moerman and Waterston 1984), extremely elevated Tc*1* copy-number and drastically reduced fitness (latter 2 features confirmed in this study), whereas CB4851 has a high transposon content (though not as elevated as BO/RW7000 and RW6999) but is thought to lack active germline transposition of Tc*1* (Nigon and Félix 2017). What evolutionary scenario would explain the active germline transposition of Tc*1* in some Bergerac sublineages and not others? Under one scenario, the wild ancestor of all Bergerac strains possessed active germline transposition of Tc*1* which has been suppressed (selection) or lost (genetic drift) in some Bergerac derivatives following laboratory domestication. A second scenario to be considered is the activation of Tc*1* germline transposition after introduction to the laboratory, sometime in the period between 1944 and 1969 (see schematic of tentative history of the Bergerac strain in Nigon and Félix 2017) followed by subsequent suppression in different Bergerac lineages. It is conceivable that laboratory selection for novel mutations resulted in the preferential retention of sublineages of Bergerac that generated more mutations due to germline Tc*1* activity. The question of how Tc*1* proliferation in the Bergerac strains was initiated is still open.

## Supplementary Material

jkac214_Supplemental_Material

jkac214_Supplemental_Table_S1

jkac214_Supplemental_Table_S10

jkac214_Supplemental_Table_S11

## Data Availability

Whole-genome sequence files are available in fastq format from the NCBI SRA database under Bioproject PRJNA801693 (https://www.ncbi.nlm.nih.gov/sra/PRJNA801693). Accessions for individual strains sequenced in this study are as follows: Bristol N2 (SRR17809361), RW7000 (SRR17809362), RW6999 (SRR17809363), and CB4851 (SRR17809364). Supplemental material is available at G3 online.
