# Supplementary material for: Bergerac strains of Caenorhabditis elegans revisited: expansion of Tc1 elements imposes a significant genomic and fitness cost
Source: G3 (Bethesda). 2022 Aug 17;12(11):jkac214. doi: 10.1093/g3journal/jkac214 (PMC9635669; doi:10.1093/g3journal/jkac214)
Supplement: jkac214_Supplemental_Material [file jkac214_supplemental_material.pdf]

Supplemental Material

Daigle *et al.* 2022. *"Bergerac Strains of C. elegans Revisited: Expansion of* Tc*1 elements Impose a Significant Genomic and Fitness Cost."*

Supplemental Table S2 Tukey–Kramer HSD method of multiple comparisons of mean productivity among pairs of strains.

	**N2**	**CB4851**	**RW6999**	**RW7000**
**N2**	–	23.15	21.39	22.08
**CB4851**	233.24*	–	22.13	22.79
**RW6999**	201.83*	31.41*	–	21.00
**RW7000**	265.26*	32.02*	63.42*	–

Absolute differences between all strain pairs are listed below the diagonal. Critical HSD values are listed above the diagonal. Comparisons are significant ($\alpha = 0.05$) if their absolute differences are greater than their critical HSD (indicated with an asterisk).

Supplemental Table S3 Tukey–Kramer HSD method of multiple comparisons of mean survivorship to adulthood among pairs of strains.

	**N2**	**CB4851**	**RW6999**	**RW7000**
**N2**	–	0.050	0.047	0.048
**CB4851**	0.072*	–	0.047	0.047
**RW6999**	0.091*	0.019	–	0.044
**RW7000**	0.149*	0.077*	0.058*	–

Absolute differences between all strain pairs are listed below the diagonal. Critical HSD values are listed above the diagonal. Comparisons are significant ($\alpha = 0.05$) if their absolute differences are greater than their critical HSD (indicated with an asterisk).

Supplemental Table S4 Tukey–Kramer HSD method of multiple comparisons of mean longevity among pairs of strains.

	N2	CB4851	RW6999	RW7000
**N2**	–	2.35	2.18	2.30
**CB4851**	3.51*	–	2.25	2.40
**RW6999**	4.97*	1.46	–	2.19
**RW7000**	3.95*	0.44	1.02	–

Absolute differences between all strain pairs are listed below the diagonal. Critical HSD values are listed above the diagonal. Comparisons are significant ($\alpha = 0.05$) if their absolute differences are greater than their critical HSD (indicated with an asterisk).

Supplemental Table S5 Tukey–Kramer HSD method of multiple comparisons of mean developmental time (hours) among pairs of strains.

	**N2**	**CB4851**	**RW6999**	**RW7000**
**N2**	-	3.87	3.56	3.68
**CB4851**	12.21*	-	3.70	3.81
**RW6999**	5.77*	6.44*	-	3.50
**RW7000**	12.85*	0.65	7.08*	-

Absolute differences between all strain pairs are listed below the diagonal. Critical HSD values are listed above the diagonal. Comparisons are significant ($\alpha = 0.05$) if their absolute differences are greater than their critical HSD (indicated with an asterisk).

Supplemental Table S6 Tukey–Kramer HSD method of multiple comparisons of mean speed (μm/s) among pairs of strains.

	**N2**	**CB4851**	**RW6999**	**RW7000**
**N2**	-	35.18	35.18	35.18
**CB4851**	12.07	-	35.18	35.18
**RW6999**	56.66*	44.59*	-	35.18
**RW7000**	125.08*	113.01*	68.43*	-

Absolute differences between all strain pairs are listed below the diagonal. Critical HSD values are listed above the diagonal. Comparisons are significant ($\alpha = 0.05$) if their absolute differences are greater than their critical HSD (indicated with an asterisk).

Supplemental Table S7 Tukey–Kramer HSD method of multiple comparisons of mean body length (μm) among pairs of strains.

	N2	CB4851	RW6999	RW7000
**N2**	-	60.23	60.23	60.23
**CB4851**	152.24*	-	60.23	60.23
**RW6999**	123.67*	28.56	-	60.23
**RW7000**	163.41*	11.17	39.73	-

Absolute differences between all strain pairs are listed below the diagonal. Critical HSD values are listed above the diagonal. Comparisons are significant (α = 0.05) if their absolute differences are greater than their critical HSD (indicated with an asterisk).

Supplemental Table S8 Tukey–Kramer HSD method of multiple comparisons of mean body area (μm^2) among pairs of strains.

	N2	CB4851	RW6999	RW7000
**N2**	-	6648	6648	6648
**CB4851**	10321*	-	6648	6648
**RW6999**	11113*	792	-	6648
**RW7000**	18813*	8492*	7700*	-

Absolute differences between all strain pairs are listed below the diagonal. Critical HSD values are listed above the diagonal. Comparisons are significant ($\alpha = 0.05$) if their absolute differences are greater than their critical HSD (indicated with an asterisk).

Supplemental Table S9 Tukey–Kramer HSD method of multiple comparisons of mean direction change (radians/*s*) among pairs of strains.

	N2	CB4851	RW6999	RW7000
**N2**	-	0.198	0.198	0.198
**CB4851**	0.099	-	0.198	0.198
**RW6999**	0.057	0.042	-	0.198
**RW7000**	0.598*	0.499*	0.541*	-

Absolute differences between all strain pairs are listed below the diagonal. Critical HSD values are listed above the diagonal. Comparisons are significant ($\alpha = 0.05$) if their absolute differences are greater than their critical HSD (indicated with an asterisk).

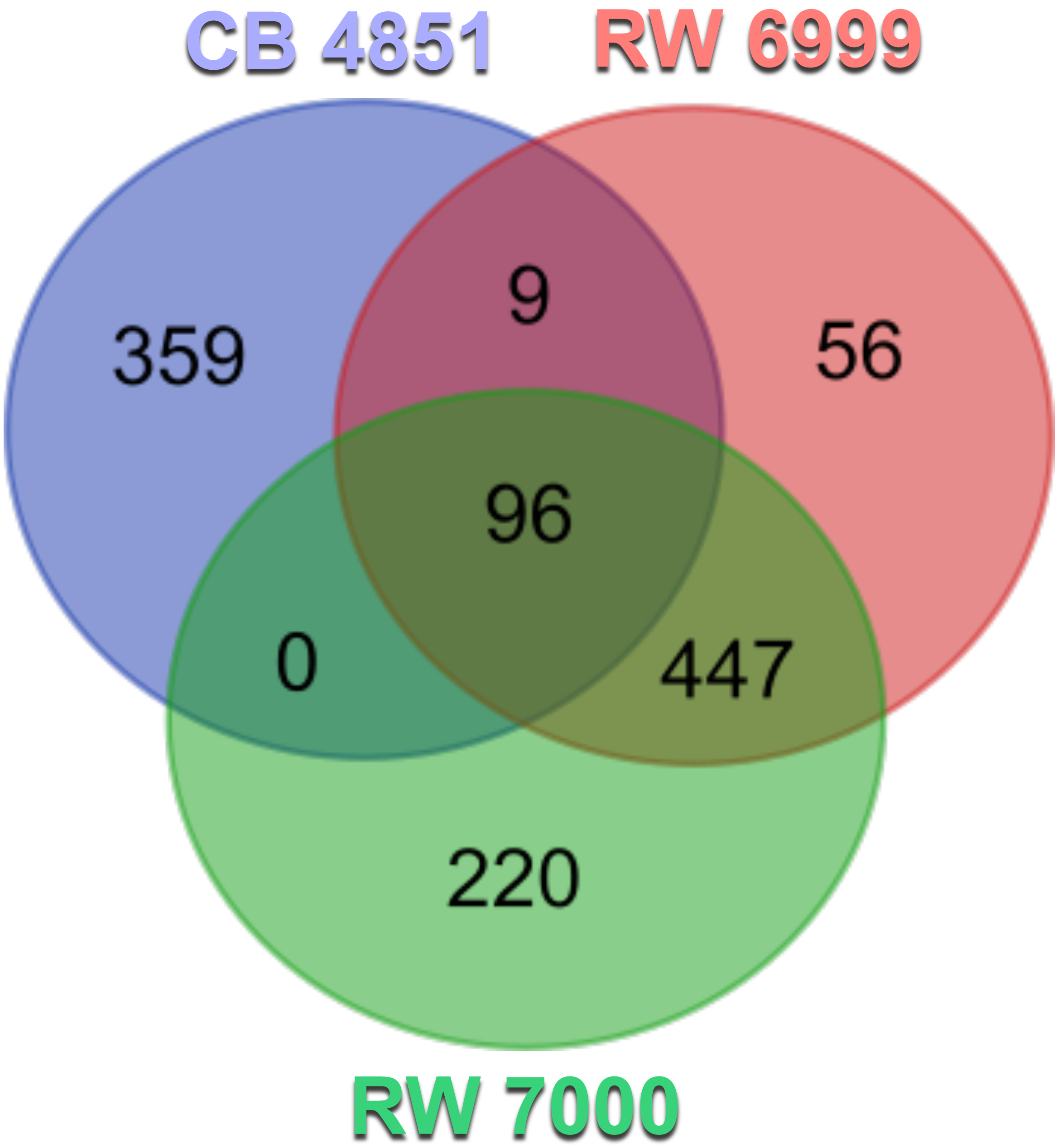

Supplemental Figure S1 Comparison of TEMP2 Tc*1* calls between the three Bergerac strains. The overlap of Tc*1* insert locations called by TEMP2 was assessed by assigning Tc*1* calls to windows (1,000 bp for non-reference calls and 100,000 bp for reference calls), and then comparing the calls for CB4851 (blue), RW6999 (red), and RW7000 (green). Although the three Bergerac strains share 96 Tc*1* inserts, the strains RW6999 and RW7000 share more insertion sites with each other than with CB4851, reflecting the unique histories of laboratory cultivation of these strains.

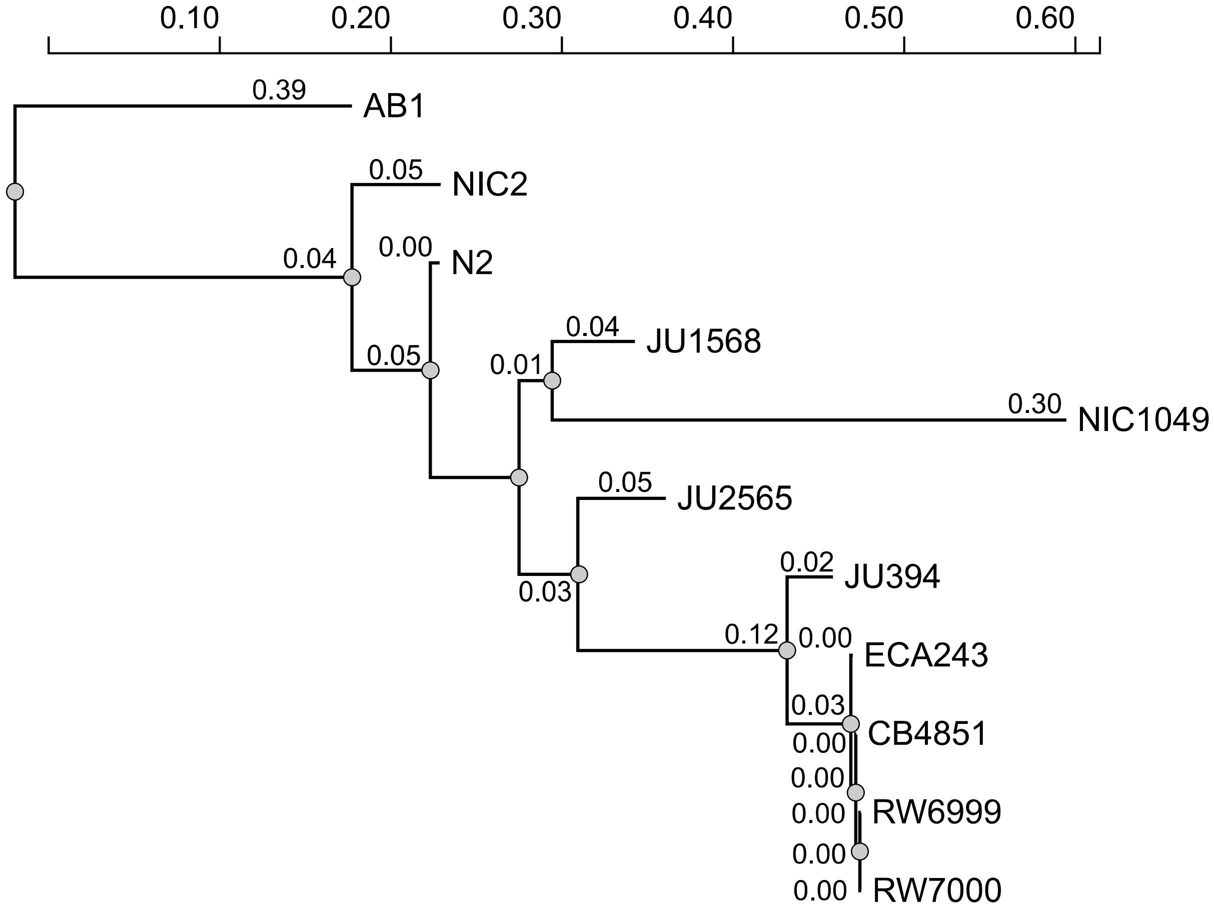

Supplemental Figure S2 Phylogenetic relationships between the three focal Bergerac strains (CB4851, RW6999, RW7000), N2 and seven other *C. elegans* natural isolates. The maximum-likelihood phylogenetic tree was created using nuclear and mitochondrial SNPs for the three Bergerac strains, the N2 strain housed in the laboratory, and the natural isolates AB1, ECA243, JU1568, JU2565, JU394, NIC1049, and NIC2. The Bergerac strains display close relationships to each other, including a previously sequenced genome of CB4851, renamed ECA243. Two strains most closely related to the Bergerac strains, JU394 and JU2565, were both isolated from France in 2016 and 2017, respectively.
